# Aging affects the in vivo regenerative potential of human mesoangioblasts

**DOI:** 10.1111/acel.12714

**Published:** 2018-02-04

**Authors:** Alessio Rotini, Ester Martínez‐Sarrà, Robin Duelen, Domiziana Costamagna, Ester Sara Di Filippo, Giorgia Giacomazzi, Hanne Grosemans, Stefania Fulle, Maurilio Sampaolesi

**Affiliations:** ^1^ Translational Cardiomyology Laboratory Stem Cell Institute of Leuven Unit of Stem Cell Research Cluster of Stem Cell and Developmental Biology Department of Development and Regeneration University of Leuven Leuven Belgium; ^2^ Department of Neuroscience, Imaging and Clinical Sciences University “G. d'Annunzio” Chieti‐Pescara Chieti Italy; ^3^ Interuniversity Institute of Myology Chieti Italy; ^4^ Human Anatomy Unit Department of Public Health, Experimental and Forensic Medicine University of Pavia Pavia Italy

**Keywords:** aging, muscular dystrophy, myogenic differentiation potential, sarcopenia, skeletal muscle and myopathies

## Abstract

Sarcopenia is the age‐related loss of muscle mass, strength, and function. Although the role of human satellite cells (SCs) as adult skeletal muscle stem cells has been deeply investigated, little is known about the impact of aging on muscle interstitial stem cells. Here, we isolated the non‐SC CD56^–^ fraction from human muscle biopsies of young and elderly subjects. The elderly interstitial cell population contained a higher number of CD15^+^ and PDGFRα^+^ cells when compared to young samples. In addition, we found that the CD56^–^/ALP ^+^ cells were well represented as a multipotent stem cell population inside the CD56^–^ fraction. CD56^–^/ALP ^+^/CD15^–^ cells were clonogenic, and since they were myogenic and expressed NG2, α‐SMA and PDGFRβ can be considered mesoangioblasts (MABs). Interestingly, elderly MABs displayed a dramatic impairment in the myogenic differentiation ability in vitro and when transplanted in dystrophic immunodeficient *Sgcb‐null Rag2‐null* γ*c‐null* mice. In addition, elderly MABs proliferated less, but yet retained other multilineage capabilities. Overall, our results indicate that aging negatively impacted on the regenerative potential of MABs and this should be carefully considered for potential therapeutic applications of MABs.

## 1 INTRODUCTION

Sarcopenia is the age‐related loss of muscle mass, strength, and function (Delbono, 2011). Muscle wasting can cause severe debilitating weakness and currently represents an important clinical problem with few solutions (Birbrair et al., 2014). Satellite cells (SCs) are the muscle‐specific stem cells, which are located between the basal lamina and sarcolemma of individual myofibers (Boldrin & Morgan, 2013). These cells participate in skeletal muscle repair in response to injury, but they are scarce and difficult to isolate (Berardi, Annibali, Cassano, Crippa, & Sampaolesi, 2014). Although it is still controversial, the number and function of SCs decline with age and correlate with decreased regeneration ability following injury or physical activity in elderly individuals (Sousa‐Victor et al., 2014; Tajbakhsh, 2013). However, few studies (Birbrair et al., 2014) have been performed to address the effect of aging on other stem/progenitor cell populations that are resident in the adult skeletal muscle, for example, interstitial cells (Malecova & Puri, 2012). Moreover, most of these studies have been performed using mouse models, while as far as we know, there is a lack of findings in humans. Interstitial cells, including pericytes and fibro/adipogenic progenitors (FAPs), can likely contribute to the reduced muscle regeneration and increased fat deposition (Birbrair et al., 2013; Uezumi, Fukada, Yamamoto, Takeda, & Tsuchida, 2010), which are hallmarks of aging. Mesoangioblasts (MABs), vessel‐associated progenitor/stem cells isolated from postnatal skeletal muscle, were described as a subset of muscle pericytes (Quattrocelli, Costamagna, Giacomazzi, Camps, & Sampaolesi, 2014). Intra‐arterial transplantation of murine (Sampaolesi et al., 2003), canine (Sampaolesi et al., 2006), and human (Dellavalle et al., 2007) MABs in animal models of muscular dystrophy demonstrated that they engrafted well and consequently improved skeletal muscle function. Human MABs have been shown to own myogenic differentiation potential in vitro, and when intra‐arterial injected into injured muscles, they gave rise to higher number of donor fibers in comparison with SCs, unable to cross the endothelial wall. Other recent studies reported that MABs resident in adult skeletal muscle are myogenic in vitro (Pierantozzi et al., 2016) and in vivo (Cossu et al., 2015; Costamagna et al., 2016). FAPs have been shown to possess an adipogenic potential in vitro and in vivo and are currently isolated in mice as Sca1^+^, or Pdgfrα^+^ (platelet‐derived growth factor receptor alpha) cells since these two populations are considered equivalent (Joe et al., 2010; Uezumi et al., 2010). However, there is no agreement on the antigen expression that characterizes human adipogenic progenitors (Webster, Pavlath, Parks, Walsh, & Blau, 1988). Nevertheless, cells with adipogenic potential have already been isolated from human skeletal muscle using CD34 (Pisani et al., 2010) or PDGFRα (Uezumi et al., 2014) as markers. Thus, PDGFRα^+^ cells could represent the human counterpart of the murine Sca1^+^ FAPS. Moreover, a recent study (Arrighi et al., 2015) described the carbohydrate adhesion molecule CD15 (3‐fucosyl‐N‐acetyl‐lactosamin) to be expressed at the surface of human muscle‐derived adipogenic progenitors. Nevertheless, there is no common consensus on markers that are able to discriminate one single population due to high heterogeneity of cells resident in the muscle interstitium. For instance, pericytes have also been shown to adopt adipogenic lineages in vitro and in vivo (Crisan et al., 2008). Indeed, *Traktuev* et al. identified a specific group of CD34^+^ adipogenic stem cells (ASCs) that possess pericyte properties, due to their expression of NG2 (neural/glial antigen 2), αSMA (alpha smooth muscle actin), and PDGFRβ (platelet‐derived growth factor receptor beta). This population has been shown to localize in vessels at the interface between endothelium and adipocytes, supporting endothelial survival (Traktuev et al., 2008). Overall, a further understanding of the effect of aging on the myogenic and adipogenic potential of human interstitial cells, for instance MABs, is required.

In the present study, we isolated and characterized human interstitial cells as the non‐SC CD56^–^ cell fraction derived from skeletal muscle biopsies of young and elderly donors. Specifically, we focused our attention on young/elderly MABs, referred here as ALP^+^ CD15^–^ cells, comparing their in vitro and in vivo differentiation capability.

## 2 RESULTS

### 2.1 Characterization and proliferation of cultured young and elderly CD56^–^ subpopulations

**Figure 1:**
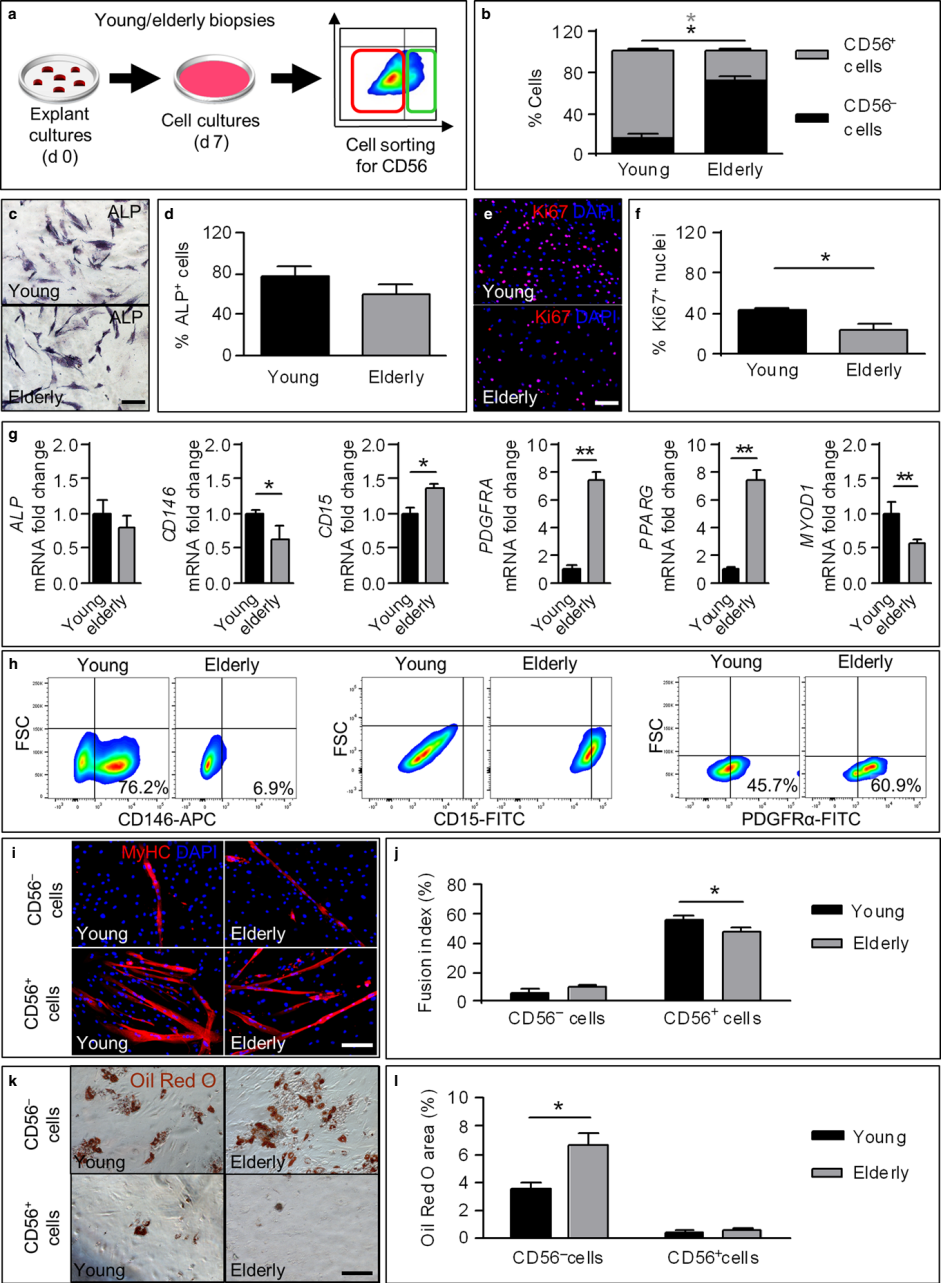
Sorting, characterization, and differentiation potential of CD56^−^ cells isolated from young and elderly donors. (a) Schematic overview of the cell sorter strategy for CD56 marker used to isolate the CD56^+^ and the CD56^−^ fractions from young and elderly subjects. (b) Graph indicating the average percentage of CD56^+^/CD56^−^ cells obtained in (a). *p <* *.05, *n *=* *5 per group. (c) Representative images of one of the young/elderly populations obtained in (a) stained with alkaline phosphatase enzymatic staining. Enzymatic activity is detected using light microscopy through the formation of an insoluble black‐purple precipitate. Scale bars: 200 μm. (d) Graph displaying the percentage of ALP ^+^ cells vs. total cells in (c). No significant differences were found regarding ALP activity between young and elderly cells (*n *=* *5 per group). (e) Immunofluorescence analysis of proliferation marker Ki67 in CD56^−^ cells 10 days after sorting. Proliferating cells were clearly detected by the nuclear staining in red. Nuclei are counterstained with DAPI. Scale bars: 200 μm. (f) Quantification of Ki67^+^ nuclei from (e). *p <* *.05, *n *=* *5 per group. (g) qRT–PCR analysis of selected adipogenic, myogenic, and mesenchymal markers. *p <* *.05, **p <* *.01, *n *=* *3 per group. (h) Facs analysis of CD146^+^, CD15^+^, and PDGFRα^+^ cells in young and elderly CD56^−^ fractions. The percentage (mean) of each marker is indicated in the lower right quadrants. Isotype antibodies were used as negative controls. (i) Representative images of CD56^+^/CD56^−^ sorted cells induced to undergo myogenic differentiation for 10 days. Cells are stained for the myogenic marker myosin heavy‐chain (MyHC, red). Nuclei are counterstained with DAPI (blue). Scale bars: 200 μm. (j) Quantification of the number of nuclei inside myotubes expressing MyHC (fusion index). *p <* *.05, *n *=* *5 per group. (k) Representative images of CD56^−^/CD56^+^ fractions induced to undergo adipogenic differentiation for 14 days. Cells are stained with Oil Red O Staining. Scale bars: 200 μm. (l) Quantification of Oil Red O area represented as % of total area. *p <* *.05, *n *=* *5 per group. For b, d, f, g, j, l, two‐tailed Student's *t* test was used and results are displayed as mean ± *SEM*

Human muscle biopsies were obtained from young and elderly donors. As expected, muscle sections from elderly subjects showed a tendency to a reduction in the cross‐sectional area of the fibers, while a significant increase in areas of fibrosis was observed (Figure S1a,b). Subsequently, CD56^+^ and CD56^–^ cells were obtained from the muscle biopsies by fluorescence‐activated cell sorter (Figure 1a). The amount of CD56^–^ fraction was significantly higher in elderly cultures (70.9% ± 6.8%) in comparison with the young ones (29.1% ± 2.9%) (Figure 1b). All CD56^+^ cells were found to be desmin^+^ by immunofluorescence analysis, while very few CD56^–^ cells showed positive signal for desmin in both young and elderly samples (Figure S2a). Alkaline phosphatase (ALP) enzymatic staining (Figure 1c,d) showed that both young and elderly CD56^–^ fractions were enriched in ALP^+^ cells, accounting for, respectively, 73.4% ± 5.6% and 56.8% ± 9.1% of the total cell amount. qRT–PCR analysis of both populations showed similar *ALP* expression (Figure 1g). In addition, young CD56^–^ cells showed a higher percentage of Ki67^+^ cells compared to elderly ones (Figure 1e,f). Moreover, the number of Ki67^+^ cells was higher in the CD56^–^ fraction compared to the CD56^+^ counterpart, both in young and in elderly samples (data not shown).

qRT–PCR analysis (Figure 1g) revealed a significant upregulation of the perivascular marker *CD146/MCAM* (melanoma cell adhesion molecule) in young CD56^–^ cells compared to their elderly counterpart. Conversely, human FAP markers *CD15* and *PDGFR*α were significantly increased in elderly samples. Accordingly, the gene encoding for the master adipogenic transcription factor peroxisome proliferator‐activated receptor gamma (*PPARG*) was higher expressed in elderly samples, while the expression of *MYOD1* gene, which encodes for the master myogenic transcriptor factor MyoD, was increased in young samples. Moreover, when comparing the CD56^–^ pool with its CD56^+^ counterpart, the expression of both *CD15* and *PDGFRA* was found significantly higher in CD56^–^ cells (Figure S2b,c).

FACS analysis confirmed a higher content of CD146^+^ cells in young CD56^–^ samples compared to the elderly CD56^–^ ones, and a higher percentage of both CD15^+^ and PDGFRα^+^ cells in the elderly CD56^–^ pool when compared to young (Figure 1h).

Taken together, these findings underlined a biased adipogenic lineage commitment in the interstitial CD56^–^ aged cells.

### 2.2 Myogenic and adipogenic differentiation potential of young and elderly CD56^–^ and CD56^+^ subpopulations

CD56^–^ cells cultured in myogenic medium for 10 days showed poor myogenic capacity compared to CD56^+^ cells, both in young and elderly cultures (Figure 1i,j). When cells were cultured in adipogenic medium, Oil Red O staining showed that CD56^–^ cells accumulated lipid droplets, while CD56^+^ cells featured very few lipid droplets (Figure 1k,l), both in young and elderly cultures. In addition, lipid droplets were also analyzed by immunofluorescence staining for perilipin (PLN1) and the percentage of PLN1^+^ cells was significantly higher in the elderly CD56^–^ fraction in contrast to its young CD56^–^ counterpart (Figure S2d,e). Moreover, no PLN1^+^ cells were observed in the CD56^+^ populations.

**Figure 2:**
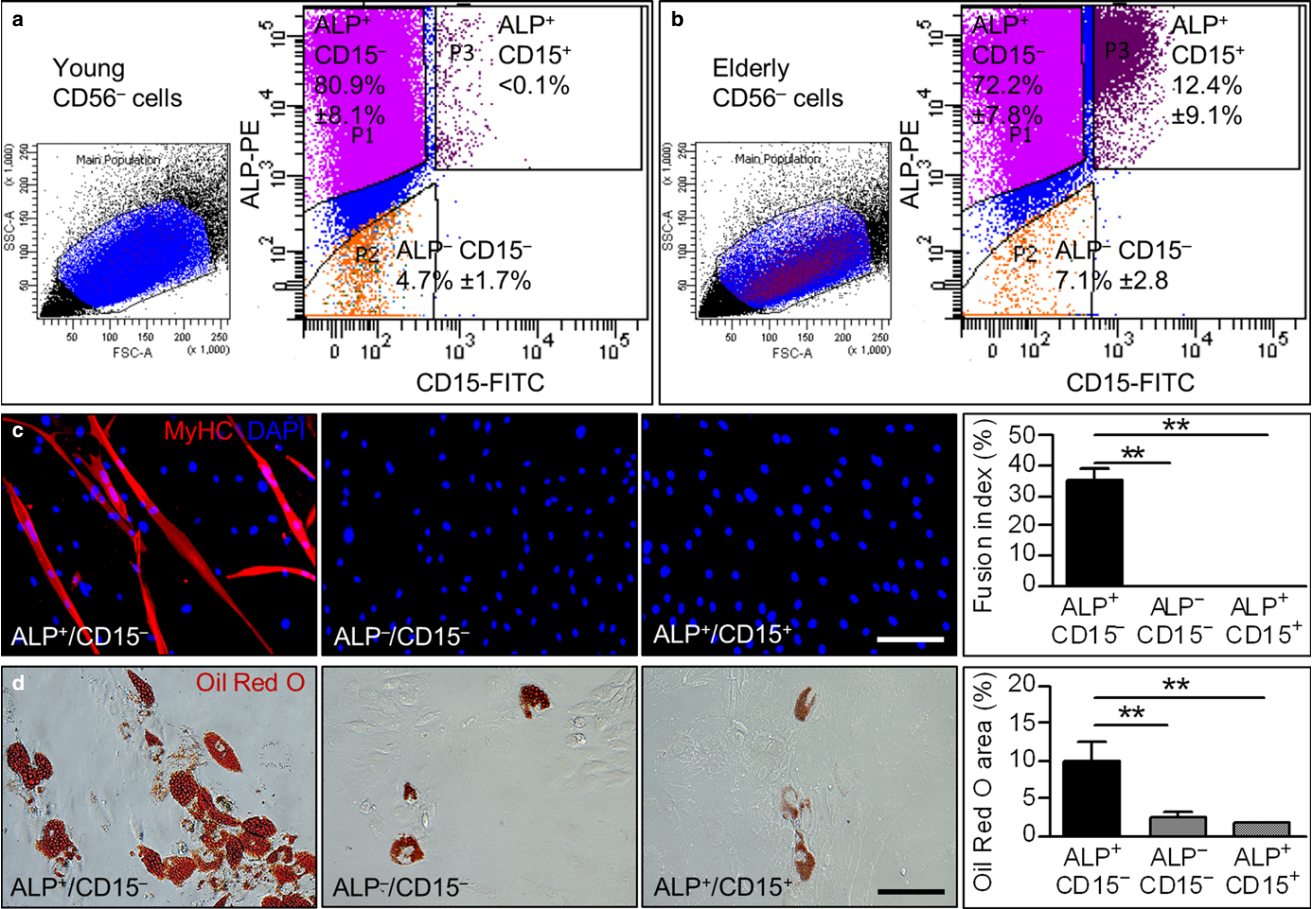
FACS cell sorting analysis and in vitro myogenic and adipogenic differentiation of CD56^−^/ALP ^+^/CD15^−^, CD56^−^/ALP ^−^/CD15^−^, and CD56^−^/ALP ^+^/CD15^+^ cells. (a, b) Examples of dot plot indicating the different subpopulations (CD56^−^/ALP ^+^/CD15^−^, CD56^−^/ALP ^−^/CD15^−^, and CD56^−^/ALP ^+^/CD15^+^) obtained by combining anti‐ALP‐PE and anti‐CD15‐FITC antibodies. The percentage of each subpopulation is represented as mean ± *SEM* (*n *=* *3 per group). (c) Immunofluorescence analysis for MyHC (red) of each cellular fraction after 10 days in myogenic medium. Nuclei are counterstained with DAPI (blue). Scale bars: 200 μm. Right panel indicates the higher fusion index in CD56^−^/ALP ^+^/CD15^−^ cells. **p <* *.01, *n *=* *3 per group. (d) Oil Red O staining of the subpopulations isolated in a and b. Scale bars: 200 μm. Right panel indicates the quantification of Oil Red O area in percentage of coverage per field, showing a larger area in CD56^−^/ALP ^+^/CD15^−^ cells. **p <* *.01, *n *=* *3 per group. For c, d, two‐tailed Student's *t* test was used and results are displayed as mean ± *SEM*

Subsequently, ALP^+^/CD15^–^ cells were isolated from CD56^–^ cells using fluorescence‐activated cell sorting. ALP^+^/CD15^–^ cells represented the widest population in the CD56^–^ fractions in our cultures, accounting for approximately 80.9% ± 8.1% and 72.2% ± 7.8% in young and elderly samples, respectively (Figure 2a,b). Interestingly, ALP^+^/CD15^+^ cells were present only in elderly CD56^–^ cells (12.4% ± 9.1%) but nearly absent in young cultures (<0.1%). Moreover, ALP^–^/CD15^–^ cells accounted for 4.7% ± 1.7% in young samples and for 7.1% ± 2.8% in elderly ones (Figure 2a,b). We also confirmed that CD15 was present in situ in muscles from elderly subjects, while almost no CD15 expression was detectable on the sections of young muscles (Figure S1c). Selected cell fractions were then subjected to myogenic and adipogenic differentiation assays. Our results showed that ALP^+^/CD15^–^ cells displayed the capacity to form both myotubes (fusion index = 34.9% ± 2.7%) and adipocytes (% Oil Red O area vs. total area = 9.9% ± 2.7), whereas ALP^+^/CD15^+^ and ALP^–^/CD15^–^ cells showed no differentiation potential to the myogenic lineage (Figure 2c) and only a limited capacity toward adipogenic commitment (Figure 2d).

Our results showed that elderly samples are more prone to adipogenic differentiation and CD56^–^/ALP^+^/CD15^–^ cells exhibit the greater plasticity.

### 2.3 Characterization of young and elderly mesoangioblasts

**Figure 3:**
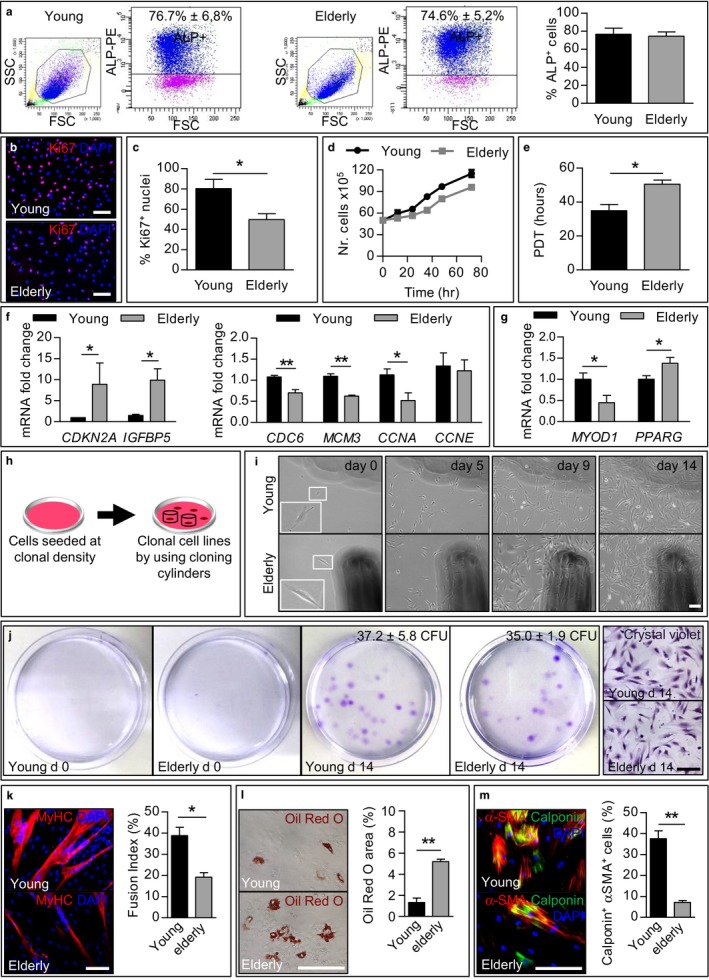
Characterization, proliferation, and mesodermal differentiation potential of young and elderly MABs. (a) Representative dot plot of fluorescence‐activated cell sorting of young/elderly ALP ^+^ cells (MABs; *n *=* *5 per group). The histogram on the right represents the percentage of the ALP ^+^ obtained. (b) Immunofluorescence analysis for the proliferation marker Ki67 (red) in young/elderly MABs. Nuclei are counterstained with DAPI. Scale bars: 200 μm. (c) Quantification of Ki67‐positive nuclei from (b). *p <* *.05, *n *=* *5 per group. (d) Growth curve of young and elderly MABs over time (*n *=* *5 per group). (e) Population doubling time of young and elderly MABs. *p <* *.05, *n *=* *5 per group. (f, g) qrt–PCR analysis for the senescence markers *CDKN2A, IGFBP5, CDC6, MCM3, CCNA*, and *CCNE* (f) and for the myogenic and adipogenic markers *MYOD1* and *PPARG* (g), respectively, in young and elderly MABs. *p <* *.05, *n *=* *3 per group. (h) Schematic overview of the generation of clonal cell lines. Young and elderly MABs were plated at clonal density (0.9 cells/cm^2^) and cultured for 14 days. Cloning cylinders were then used to obtain colonies from single cells. (i) Cell morphology of young and elderly clonal cell lines from day 0 (left panels; inserts show an enlarged area containing an example of single cell that gives rise to a colony) until day 14 (right panels; >100 cells/colony). (j) Representative images of the colony formation assay (*n = 5*). Crystal violet staining shows the colonies formed at day 0 and 14 after clonal density seeding. The number of colonies formed is indicated in the upper right part of each image. Right panels show magnifications of representative young and elderly colonies; scale bars: 200 μm. (k) Representative images of immunofluorescence analysis for the myogenic marker MyHC (red) on clonal cell lines from young and elderly MABs cultured for 10 days in myogenic medium. Nuclei are counterstained with DAPI (blue). Scale bars: 200 μm. The graph indicates the quantitative analysis of the number of nuclei inside myotubes expressing MyHC (fusion index). *p <* *.05, *n *=* *6 clones per group. (l) Representative images of Oil Red O staining on clonal cell lines from young and elderly MABs cultured for 14 days in adipogenic medium. Scale bars: 200 μm. The graph indicates the quantification of Oil Red O area in percentage of coverage per field. **p <* *.01, *n *=* *6 clones per group. (m) Representative images of immunofluorescence analysis for the smooth muscle markers calponin (green) and α‐SMA (red) on clonal cell lines from young and elderly MABs cultured for 7 days in smooth muscle differentiation medium. Nuclei are counterstained with DAPI (blue). Scale bars: 200 μm. The graph indicates the quantitative analysis of the percentage of differentiated cells expressing both smooth muscle markers. **p <* *.01, *n *=* *6 clones per group. For a, c–g, k–m, two‐tailed Student's *t* test was used and results are displayed as mean ± *SEM*

Next, we characterized ALP^+^/CD15^–^ cells, termed from here on as MABs, since they expressed NG2 and PDGFRβ (Figure S3c) and they exhibited a stronger myogenic commitment compared to the other interstitial populations (Figure 2c). ALP^+^/CD15^–^ cells were directly isolated from fresh muscle biopsies (Table S1) and they were similarly represented in young and elderly samples (Figure 3a). No differences in morphology were observed between the two populations (Figure S3a). In addition, both young and elderly MABs were positive for ALP and α‐SMA markers (Figure S3a,b), highly positive for the mesenchymal marker CD90 (Thy1, >90%), and negative for the endothelial lineage marker CD31 (platelet endothelial cell adhesion molecule, <2%) (Figure S3c). Elderly MABs showed less PDGFRβ^+^ and NG2^+^ cells compared to young samples (34.3% vs. 83.7% and 23.1% vs. 64.3%, respectively) by flow cytometric analysis (Figure S3c). Moreover, elderly MABs showed higher expression of two fibrosis markers, collagen type III alpha 1 chain (*COL3A1*) and metalloproteinase inhibitor 1 (*TIMP1*), when compared to young MABs (Figure S3d).

Interestingly, young and elderly MABs exhibited significant differences in their proliferation capacity, assessed by Ki67 immunofluorescence analysis (Figure 3b,c). The growth curve (Figure 3d) and the population doubling time (Figure 3e) also confirmed the impairment in proliferation associated with aging. This disparity could be explained by the differential gene expression of two important markers of cellular senescence: cyclin‐dependent kinase inhibitor 2A (*CDKN2A* or *p16* ^*INK4a*^) and insulin‐like growth factor binding protein 5 (*IGFBP5;* Figure 3f). qRT–PCR analysis of genes downstream the p16^INK4a^/Rb/E2F cascade, that are, cell division cycle 6 (*CDC6*), minichromosome maintenance complex component 3 (*MCM3*), cyclin A2 (*CCNA*), and cyclin E1 (*CCNE*), confirmed that aged MABs presented a dysregulation in senescence‐related genes (Figure 3f, left panel).

These results showed that MABs can be easily isolated from young and old muscle samples and that elderly cells display a reduced cell proliferation associated with a senescent‐like behavior.

### 2.4 Mesodermal capability of young and elderly mesoangioblasts

Next, we tested the mesodermal commitment of young and elderly MABs. Young MABs showed significantly higher expression of *MYOD1* and lower expression of the adipogenic marker *PPARG* when compared to elderly MABs by qRT–PCR analyses (Figure 3g). Consistently with the previous results, young MABs cultured in myogenic differentiation medium fused to form multinucleated myotubes that expressed MyHC, as assessed by immunofluorescence analysis (Figure S3f). Although elderly MABs in coculture with C2C12 cells gave rise to hybrid myotubes, they formed alone significantly fewer and smaller MyHC^+^ myotubes compared to young MABs (Figure S3e,f).

When cultured in adipogenic differentiation medium, Oil Red O staining revealed a significantly wider area of lipid droplets in the elderly population compared to the young population (Figure S3g).

Additionally, we assessed the ability of MABs to undergo smooth muscle differentiation. We observed that MABs derived from young and elderly donors became bigger, flatter, and with more apparent cytoplasm content from day 4 onwards of smooth muscle differentiation (data not shown). At day 7, differentiated cells resulted to be positive for both the smooth muscle markers α‐SMA and calponin as revealed by immunofluorescence analysis (Figure S3h). However, differentiated elderly MABs presented a significantly lower percentage of double‐positive cells (α‐SMA^+^/calponin^+^) compared to young MABs (Figure S3h).

Differentiation potential experiments were then repeated using clonal cells to confirm the multipotency of MABs. Cells were seeded at clonal density (0.9 cells/cm^2^), and colonies originated from a single cell were isolated using cloning cylinders after 10–14 days (Figure 3h,i). No difference was observed in the colony‐forming unit assay (Figure 3j) and all clones analyzed exhibited tripotency, the ability to give rise to the three different lineages tested. Upon myogenic induction, clonal cells from young MABs featured a significant higher fusion index compared to clones from elderly MABs (Figure 3k). Conversely, elderly clones cultured in adipogenic differentiation medium presented a significant wider area of lipid droplets compared to young clones (Figure 3l). Regarding smooth muscle differentiation, the presence of α‐SMA^+^/calponin^+^ cells was found significantly higher in young clonal cells when compared to elderly ones (Figure 3m).

To uncouple cell proliferation/density effect and myogenic differentiation potential, aged MABs were plated at higher cell densities (1.5‐fold and 2‐fold) compared to the more proliferative young MABs (1‐fold). Subsequently, cells were induced to skeletal muscle lineage. Interestingly, we observed an increased fusion index in aged cells seeded at 2‐fold density compared to aged cells seeded at lower densities (Figure S4a,b). However, young cells still presented a higher fusion index compared to elderly cells under any observed conditions. qRT–PCR analysis for the myogenic markers *MYOG*,* MYH1*, and *ACTA1* further supported these data (Figure S4c). Thus, the impairment of elderly cells was not exclusively due to differences in cell proliferation and therefore density.

Considering recent results showing spontaneous myogenic capability specific for CD146^+^ cells from human muscles, we also isolated ALP^+^/CD146^–^ and ALP^–^/CD146^+^ cells from CD56^–^ cells using fluorescence‐activated cell sorting (Figure S5a,b). While after five passages cells were still positive for CD146, the number of cells with ALP enzyme activity decreased (Figure S5c,d). In our conditions, we observed that the subset of cells ALP^+^/CD146^–^ was endowed with more myogenic potential compared to the ALP^–^/CD146^+^ cell fraction (Figure S5e,f).

Taken together, these results showed that aging leads to a decline of multipotency in MABs and suggested that an adipogenic alternative program might be more pronounced in elderly MABs.

### 2.5 Young and elderly mesoangioblasts in vivo myogenic potential

**Figure 4:**
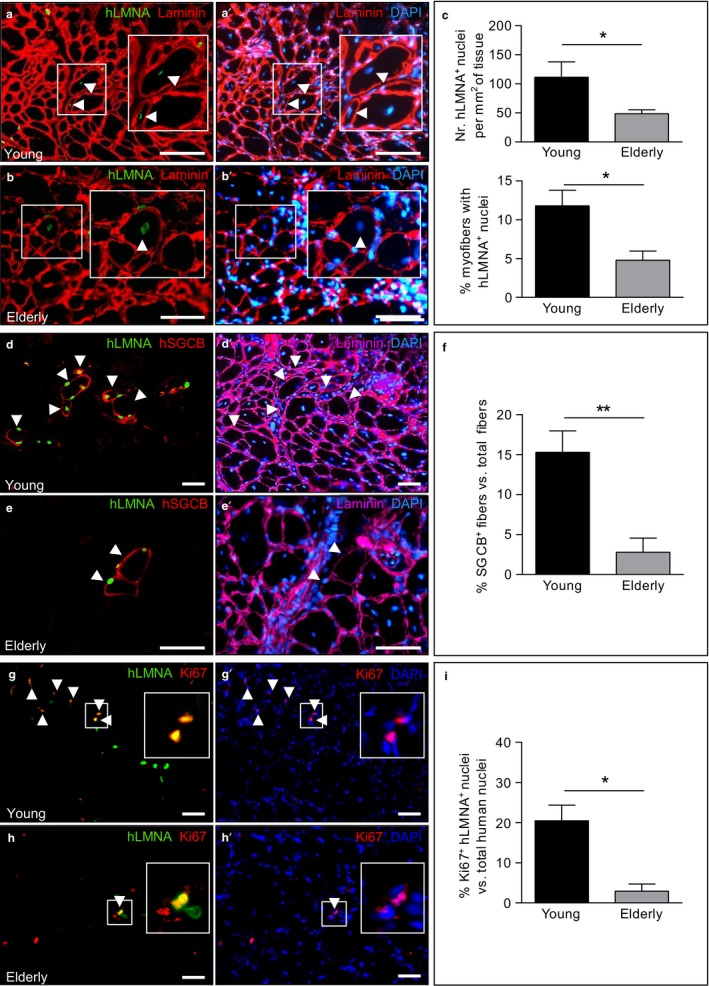
In vivo contribution to muscle regeneration of young and elderly MABs injected into TA muscles of *Sgcb‐null Rag2‐null yc‐null* mice. (a, b) Immunofluorescence analysis for hLMNA (green) and laminin (red) in transplanted TA muscles. Centronucleated fibers showed positive staining for hLMNA (green) in muscles transplanted with young (a) and elderly (b) MABs. Enlarged areas with positive human nuclei (arrowheads) are represented in the insets. Nuclei are counterstained with DAPI (blue). Scale bars: 100 μm. (c) Quantification of human nuclei hLMNA ^+^ per mm^2^ of tissue (upper panels) and quantification of the percentage of hybrid myofibers containing human nuclei vs. total myofibers (lower panel) in injected muscles with young and elderly MABs. *p <* *.05, *n *=* *4 per group. (d, e) Immunofluorescence analyses for β‐sarcoglycan (SGCB; red), human LAMIN A/C (hLMNA; green) and laminin (cyan) in muscles injected with young (d) and elderly (e) MABs. Arrowheads show SGCB ^+^ fibers. Nuclei are counterstained with DAPI (blue). Scale bars: 100 μm. (f) Percentage of SGCB ^+^ fibers vs. total fibers in injected muscles with young and elderly MABs. **p <* *.01, *n *=* *4 per group. (g, h) Immunofluorescence analysis for hLMNA (green) and Ki67 (red) in transplanted muscles with young (g) and elderly (h) MABs. Arrowheads show human proliferating cells positive for both markers. Enlarged areas with double‐positive proliferating human cells are shown. Nuclei are counterstained with DAPI (blue). Scale bars: 100 μm. (i) Quantification of human cells positive for Ki67 in muscles injected with young and elderly MABs. *p <* *.05, *n *=* *4 per group. For c, f, i, two‐tailed Student's *t* test was used and results are displayed as mean ± *SEM*

To test the myogenic potential of human MAB populations in vivo, young and elderly MABs were injected in the tibialis anterior (TA) of *Sgcb‐null Rag2‐null* γ*c‐null* mice. After 3 weeks, MAB engraftment was confirmed by immunofluorescence analysis for human‐specific lamin A/C (hLMNA) (Figures 4a,b and S6a,b). Human nuclei derived from young and elderly donors were distributed inside centronucleated fibers (Figure 4a,b) and outside the basal lamina in the interstitial space (Figure S6a,b). In addition, rare young MABs were also detected below the basement membrane in satellite cell‐like position (Figure S6c) and expressed PAX7 (Figure S6d). The amount of engrafted donor cells was found significantly higher in the young MAB‐injected TA muscles compared to the elderly MAB‐injected TA muscles (Figure 4c, upper panel). Moreover, the number of hybrid fibers (resulting from the fusion of human nuclei with host fibers) was higher in muscles injected with young MABs when compared to muscles injected with elderly MABs (Figure 4c, lower panel).

**Figure 5:**
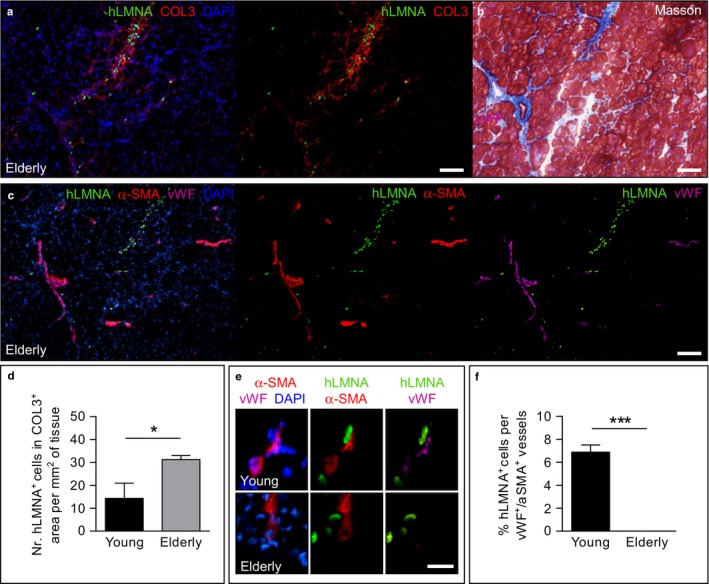
In vivo contribution to connective tissue of young and elderly MABs injected into TA muscles of *Sgcb‐null Rag2‐null yc‐null* mice. (a) Immunofluorescence analysis for hLMNA (green) and collagen type III (COL3; red) in elderly MAB‐injected muscles. (b) Masson's trichrome staining in a serial section of (a) showing connective tissue (blue) localized in the COL3^+^ area observed in (a). (c) Immunofluorescence analysis for hLMNA (green), α‐SMA (red), and von Willebrand factor (vWF, purple) in a serial section of (a). For (a–c)**,** scale bars: 200 μm. (d) Quantification of the number of human nuclei in the COL3^+^ area per mm^2^ of tissue. *p <* *.05, *n *=* *4 per group. (e) Immunofluorescence analysis for hLMNA (green), α‐SMA (red) and vWF (purple) in young and elderly MAB‐injected muscles. Young MABs are found associated with α‐SMA ^+^/vWF ^+^ vessels, in contrast to elderly MABs. Scale bars: 50 μm. For a, c, e, nuclei are counterstained with DAPI (blue). (f) Quantification of the percentage of human nuclei in the vWF ^+^/α‐SMA ^+^ vessels vs. the total number of human nuclei per field. ***p <* *.001, *n *=* *4 per group. For d, f, two‐tailed Student's *t* test was used and results are displayed as mean ± *SEM*

Co‐immunostaining for β‐sarcoglycan (SGCB), hLMNA, and laminin showed that human nuclei (hLMNA^+^) formed hybrid SGCB^+^ fibers in muscles injected with young and elderly MABs, demonstrating a direct contribution of MABs to the restoration of the SCGB expression (Figure 4d,e). Clear clusters of SCGB^+^ fibers were observed in muscles injected with young MABs in contrast to rare SCGB^+^ fibers derived from aged MABs (Figure 4d–f). The number of hLMNA/Ki67 double‐positive cells was significantly higher in muscles injected with young MABs compared to those injected with elderly ones (Figure 4g–i). hLMNA^+^ elderly MABs accumulated in collagen type III (COL3)‐positive areas of transplanted muscles, suggesting that they can be involved in the fibrosis process (Figure 5a,b,d). Immunofluorescence analysis in serial sections unveiled that the connective tissue areas enriched with hLMNA^+^ elderly cells did not show vessels double positive for the endothelial marker von Willebrand factor (vWF) and the smooth muscle marker α‐SMA (Figure 5c,e,f). In contrast, less abundant young MABs present in the COL3^+^ connective tissue (Figure 5d) were found closely associated with vWF^+^/α‐SMA^+^ vessels (Figure 5e,f).

**Figure 6:**
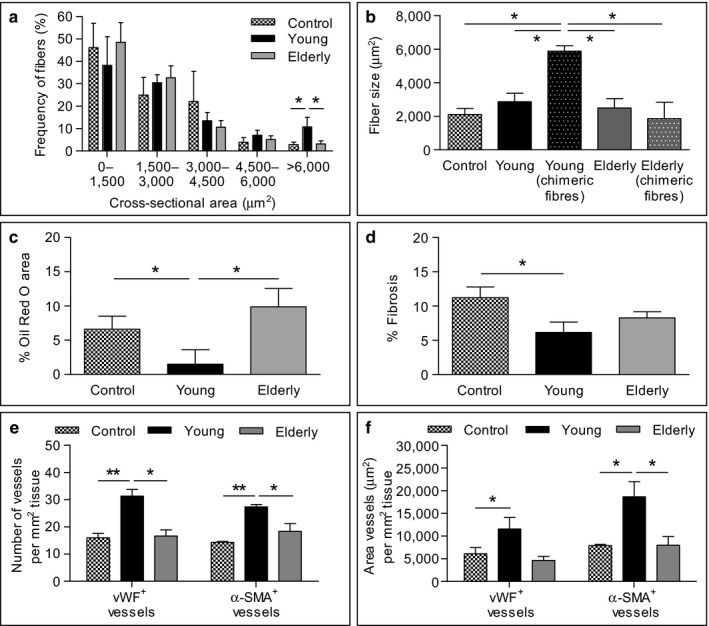
Histological and morphometric analyses of the TA muscles of *Sgcb‐null Rag2‐null yc‐null* mice injected with young and elderly MABs and angiogenic potential of young/elderly MABs. (a) Quantitative frequency distribution analysis of the cross‐sectional area of fibers in the hematoxylin and eosin staining (see Figure S7a) in injected muscles with young or elderly MABs vs. uninjected muscles. *p <* *.05, *n *=* *3 per group. (b) Quantification of the fiber size (cross‐sectional area) of muscles injected with young and elderly MABs vs. uninjected muscles. Chimeric fibers containing human nuclei in the injected muscles quantified separately. *p <* *.05, *n *=* *3. (c) Quantification of the percentage of fibrotic areas observed in the Masson's trichrome staining (Figure S7b) in injected muscles with young and elderly MABs vs. uninjected muscles. *p <* *.05, *n *=* *3 per group. (d) Quantification of the Oil Red O (see Figure S7c) areas represented as percentage of coverage per field in injected muscles with young and elderly MABs vs. uninjected muscles. *p <* *.05, *n *=* *3 per group. (e, f) Quantification of the number (e) or area (f) of vessels vWF ^+^ and α‐SMA ^+^ in injected muscles with young and elderly MABs vs. uninjected muscles (Figure S7e,f). *p <* *.05, *p *<* *.01, *n *=* *3 per group. For a–f, one‐way ANOVA was used and results are displayed as mean ± *SEM*

Morphometric analyses in the young MAB‐injected TA muscles revealed a shift in the distribution of fiber sizes toward larger fibers compared to muscles injected with elderly MABs and controls (Figures 6a,b and S7a). Indeed, chimeric fibers in muscles injected with young MABs displayed a significantly higher cross‐sectional area compared to host fibers, while this was not observed when elderly MABs were injected (Figure 6b). Moreover, Masson's trichrome staining showed significantly decreased areas of muscle fibrosis in muscles injected with young MABs in comparison with control muscles (Figures 6c and S7b). Finally, Oil Red O staining revealed that fatty deposition in muscles injected with young MABs was significantly reduced when compared to those injected with aged MABs and controls (Figures 6d and S7c). Rare elderly MABs were detected in Oil Red O^+^ areas (Figure S7d), while no young MABs were distinguished.

Additionally, to study the angiogenic potential of MABs, immunofluorescence analyses for hLMNA in combination with vWF (Figure S7d) or with α‐SMA (Figure S7e) were performed. Young MABs were found integrated into blood vessels (Figure S7d,e). However, no colocalization was observed in muscles injected with elderly MABs (Figure S7d,e). Moreover, muscles injected with young MABs presented a significantly increased number and area of vWF^+^ and α‐SMA^+^ vessels compared to control muscles (Figure 6e,f).

Our results showed that aging negatively influences the in vivo mesodermal potential of MABs and their paracrine effect when injected in dystrophic *Sgcb‐null Rag2‐null* γ*c‐null* mice.

## 3 DISCUSSION

Despite several reports on satellite cell characterization in aged mice and humans, little is known about elderly human muscle interstitial cells. In the majority of these studies, myogenic stem/progenitors were isolated on the basis of CD56 expression, known as neural cell adhesion molecule (NCAM), and present in human satellite cells, proliferating myoblasts and fibers undergoing regeneration (Sinanan, Hunt, & Lewis, 2004). For these reasons, in our study we characterized the muscle interstitial cells from young and elderly subjects defined as the CD56^–^ cell counterpart. Recently, PDGFRα (Uezumi et al., 2014) and CD15 (Arrighi et al., 2015; Laurens et al., 2016; Lecourt et al., 2010) have emerged as markers for cells endowed with a fibro/adipogenic potential in human skeletal muscles. Elucidating the interstitial cell types that directly participate in fat deposition during aging is relevant to clarify the mechanisms related to muscle homeostasis in elderly subjects. Here, we showed that the number of PDGFRα^+^ and CD15^+^ cells was increased in skeletal muscle of elderly subjects compared to young subjects. PDGFRα is expressed in several cell types, including pericytes, mature adipocytes, fibroblasts, mesenchymal/progenitor stem cells, and cardiac progenitors (Farahani & Xaymardan, 2015). However, the origin of CD15^+^ cells is still unclear and the role of CD15 in adipogenesis, as well as a direct relationship between CD15^+^ cells and PDGFRα^+^ cells, is not yet fully understood. Interestingly, in the current study, sorted CD15^+^ cells co‐expressed ALP, suggesting that CD15^+^ cells might derive from the pericyte lineage or that CD15^+^ cells and ALP^+^ cells might have a common mesodermal origin. ALP^+^/CD15^+^ cells retained a very modest adipogenic potential, therefore, we hypothesized that this population was not equivalent to the FAP population previously described (Arrighi et al., 2015). Indeed, the population we isolated could likely represent a subpopulation or a transdifferentiated population of the one previously reported (Arrighi et al., 2015). Studies aiming to characterize the muscle‐derived stem cells showed inconsistent results, likely due to the isolation protocols used in different laboratories (Bareja et al., 2014; Chirieleison, Feduska, Schugar, Askew, & Deasy, 2012; Zheng et al., 2012). However, CD15^+^ cells were observed in situ only in the muscle biopsies from elderly subjects (according to FACS data) and were not detected in young healthy muscle biopsies as already reported (Agley, Rowlerson, Velloso, Lazarus, & Harridge, 2013). Interestingly, *Pisani* et al. reported that the percentage of CD15^+^ cells isolated from human biopsies of subjects affected by myopathies was higher compared to those obtained from healthy subjects (Pisani et al., 2010). These data seemed to support our results, since sarcopenia shares similar features with dystrophic muscles, that are, muscle loss, increased fibrosis, and fatty deposition.

Mesoangioblasts are good candidates to investigate the regeneration impairment observed in elderly subjects, since they own a multilineage differentiation potential (Azzoni et al., 2014). Thus, we performed a clonal analysis of ALP^+^ MABs isolated from samples of young and elderly donors. We showed that both young and elderly single clones exhibited multipotency that could give rise to myogenic and adipogenic lineages. Accordingly, the multipotency of different interstitium‐derived stem cells has also been demonstrated in other studies (Tamaki et al., 2007). Our data showed for the first time that in vitro skeletal and smooth muscle differentiation of elderly MABs was drastically impaired when compared with young MABs. Surprisingly, the ALP^+^/CD146^−^ MAB fraction in our study was highly myogenic both in young and elderly samples. Indeed, this finding seems to contradict recent published results showing that myogenic cells were enriched in the CD146^+^/ALP^−^ cell fraction (Sacchetti et al., 2016). However, due to the small size of the biopsies for our experimental setting, enzymatic digestion and subsequent cell sorting were not feasible. For this reason, explant culture strategy was adopted. In addition, we should consider the high heterogeneity in surface markers of muscle stem cells (Chapman et al., 2013) that can be affected by different cell culture conditions (Roobrouck et al., 2011).

Several studies already demonstrated the capacity of MABs to differentiate in vivo (Costamagna et al., 2016; Quattrocelli et al., 2014), however, whether aging could affect this capability was unexplored. Thus, we tested the myogenic potential of young and elderly MABs. We found that both young and aged human MABs directly contributed to muscle regeneration fibers and to β‐sarcoglycan restoration when transplanted in *Sgcb‐null Rag2‐null* γ*c‐null* mice, although aged MABs participated at less extent. In addition, dystrophic muscles injected with young human MABs presented a higher frequency of fibers with larger cross‐sectional area, reduced fibrosis, and fat accumulation compared to muscles injected with elderly cells. Moreover, the increased cross‐sectional area of chimeric fibers in young MAB‐injected muscles suggested a direct contribution of donor cells rather than a paracrine effect. Consistent with our results, another study demonstrated that piglet muscle‐derived pericytes exhibited a greater myogenic and vasculogenic potential when compared with adult pig pericytes (Fuoco et al., 2014). Nevertheless, a recent study has claimed that murine pericytes were not able to differentiate into other cellular lineages in vivo (Guimaraes‐Camboa et al., 2017). However, Tbx18 tracer used in this study showed that ∼10% of pericytes were not labeled in the Tbx18CreERT2/TdTomato model, which suggests that a subpopulation of pericytes was not taken into account. In addition, Tbx18‐positive cells were not detected in several organs including liver, pancreas, and intestine where pericytes do exist (Birbrair et al., 2017). Thus, this study is not conclusive and requires further investigation.

Young human MABs transplanted in *Sgcb‐null Rag2‐null* γ*c‐null* mice were more proliferative than elderly MABs at day 21 postinjection. These data correlated with an increase in senescence markers (Bernet et al., 2014; Sousa‐Victor et al., 2014) including the cell cycle inhibitor and master regulator of senescence *p16* ^*INK4a*^; IGFBP5, and other downstream genes in the p16^INK4a^/Rb/E2F senescence pathway of elderly MAB cultures. Thus, the limited regenerative potential of elderly MABs could be likely due to their in vivo reduced proliferation, linked with decreased migration and fusion with host fibers (Riederer et al., 2012). Moreover, our study showed an inability of young environment (3‐month‐old mice) to fully reverse the aged phenotype in ALP^+^/CD15^−^ cells. Conversely, parabiotic pairing studies showed that the exposure to a youthful serum rejuvenated and rescued the differentiation capability of murine aged satellite cells (Conboy et al., 2005). With the limitation of culture conditions that can affect surely our results, we can speculate that cell autonomous intrinsic changes due to aging affect MABs behavior in vivo and cannot be reversed by the young environment.

Mice treated with young MABs showed a reduced fatty deposition suggesting a paracrine effect of donor cells. In this sense, several molecular cues have been shown to be involved in this mechanism and most of them are members of the transforming growth factor beta (TGFβ) superfamily (Tsurutani et al., 2011) and the wingless‐type mouse mammary tumor virus (MMTV) integration site family members (WNT) (Laudes, 2011). In this direction, the secretome of human MABs should be investigated in further studies, as already partially reported for murine MABs (Galli et al., 2005).

Moreover, several transplanted elderly MABs directly contributed to fibrotic areas and expressed higher levels of fibrotic markers when compared to young MABs. In contrast to elderly MABs, young donor cells in the collagen type III areas were associated with vessels. Thus, we hypothesize that young MABs could induce a pro‐angiogenic effect rather than contributing to fibrotic tissue. Further investigations also confirmed that transplanted young MABs were able to promote vascularization by increasing the area and the number of α‐SMA^+^ vessels together with an increase in vWF^+^ vessels. We supposed that this beneficial effect could mainly be due to both a partial direct effect and paracrine effect of MABs as previously observed in other in vivo studies (Galvez et al., 2009). In contrast, MABs derived from elderly donors did not promote revascularization in vivo. Thus, aging negatively affects the paracrine potential of MABs, as also observed in satellite cell transplantation studies (Rhoads et al., 2013).

In conclusion, our study highlighted a paramount effect of aging on human MABs. In addition to the cell‐intrinsic changes in the differentiation potential of muscle stem cells, external cues, niche stimuli, and circulating factors should be considered in the design of therapeutic strategies to ensure successful and efficacious treatment of sarcopenia.

## 4 EXPERIMENTAL PROCEDURES

### 4.1 Participant enrollment

Human muscle samples were derived from *Vastus Lateralis* muscle biopsies using the tiny percutaneous needle biopsy (TPNB) (Pietrangelo et al., 2011). Young and elderly healthy untrained subjects underwent voluntary biopsies. All these subjects provided written informed consent before participating in the present study. This study was approved by the Ethics Committee for Biomedical Research, University of Chieti (PROT 1884 COET), and complied with the Declaration of Helsinki (as amended in 2000). The criteria for the selection were normal ECG, blood pressure, absence of metabolic, cardiovascular, chronic bone/joint, and muscular diseases. Information about the age and the gender of subjects can be found in the supplementary information (Appendix S1).

### 4.2 Isolation and culture of human muscle‐derived cells

Human muscle‐derived cells were isolated from biopsies as previously described (Fulle et al., 2005). Cells were grown in Ham's F‐10 nutrient mix (Life Technologies) supplemented with 10% fetal bovine serum (FBS; Hyclone, South Logan, UT, USA), 1% glutamax (Life Technologies, Carlsbad, CA, USA) and 1% penicillin–streptomycin (P/S; Life Technologies). Cells were expanded in 100‐mm dishes at 37°C in a 5% CO_2_ incubator. The medium was changed every 2–3 days. To further expand the cells, they were detached at 80% confluence by the addition of phosphate‐buffered saline (PBS; Life Technologies) containing 0.25% trypsin–EDTA (Life Technologies) and they were replated at a seeding density of 3.5×10^3 ^cells/cm^2^. After several passages, the CD56^+^ cells and CD56^−^ cells were obtained and cultured in the above‐mentioned conditions.

### 4.3 Culture of human mesoangioblasts

Human MABs were cultured on collagen‐coated dishes (Sigma‐Aldrich, St. Louis, MO, USA) in Iscove's modified Dulbecco's media (IMDM; Life Technologies), 10% heat‐inactivated FBS (Sigma), 1% P/S, 1X glutamax, 1% sodium pyruvate (Life Technologies), 1% nonessential amino acids (Life Technologies), 0.2% 2‐mercaptoethanol (Sigma‐Aldrich), 5 ng/ml human recombinant fibroblast growth factor‐basic 2 (FGF‐2, PeproTech, Rocky Hill, NJ, USA), and 1x insulin–transferrin–selenium (ITS; Life Technologies). During expansion, medium was changed every 2–3 days.

For clonal analysis, cells from young and elderly donors were seeded at clonal density (0.9 cells/cm^2^) and cultured for 10–14 days. Cloning cylinders of 6 mm (Corning, NY, USA) were used to isolate colonies originated from a single cell, and cells were subsequently further expanded.

### 4.4 MAB transplantation in dystrophic mice

Intramuscular injections of 2.5 × 10^5^ human MABs were performed in the TA of 3‐month‐old *Sgcb‐null Rag2‐null* γ*c‐null* mice, available at KU Leuven SPF animal care facility, Belgium (*n *=* *6). MABs from three different donors at P1‐P2 were used. Uninjected limbs were used as controls. After 21 days, mice were sacrificed and muscles were snap‐frozen in liquid nitrogen‐cooled isopentane and then stored at −80°C for further analysis. The samples were then cut transversally in 7‐μm sections using a cryostat machine (Leica, Wetzlar, Germany). Immunofluorescence analyses were performed to study the tissue. Mouse procedures were performed according to the guidelines of the Institutional Animal Care and Use of KU Leuven, under the approved project with protocol code ECD N°P095/2012 issued by the *Ethische Commissie Dierproeven* of the KU Leuven.

## AUTHORS' CONTRIBUTIONS

AR participated in conception and design, collection and assembly of data, data analysis and interpretation, and manuscript writing; EM participated in collection and assembly of data, data analysis and interpretation, and manuscript writing; RD participated in collection and assembly of data; DC contributed to collection and assembly of data; ESDF contributed to collection and assembly of data; GG involved in collection and assembly of data; HG contributed to collection of data; SF participated in conception and design, data analysis and interpretation, and final approval of the manuscript; MS participated in conception and design, data analysis and interpretation, manuscript writing, and final approval of the manuscript.

## CONFLICT OF INTEREST

None declared.
