# Supplementary material for: Aging affects the in vivo regenerative potential of human mesoangioblasts
Source: Aging Cell. 2018 Feb 4;17(2):e12714. doi: 10.1111/acel.12714 (PMC5847873; doi:10.1111/acel.12714)
Supplement: Supplementary file 1 [file ACEL-17-e12714-s001.pdf]

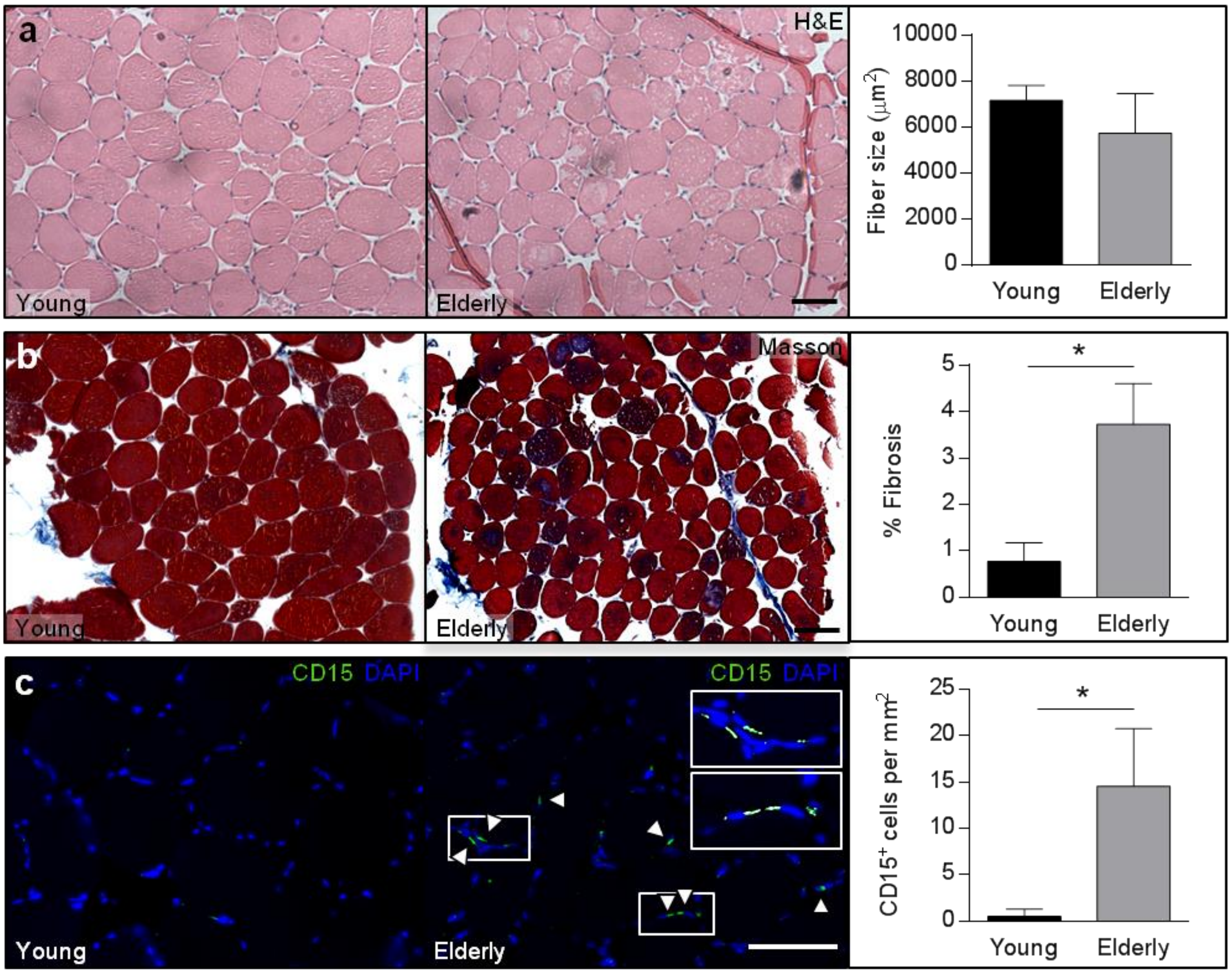

FIGURE S1 Muscles of elderly donors showing signs of sarcopenia. (a, b) Representative images of *Vastus Lateralis* muscles obtained from young and elderly donors stained for hematoxylin and eosin (a) or Masson's trichrome (b). Scale bars: 200 µm. In the right panels, graphs indicate the average of the fiber size (a) and the percentage of fibrosis areas (b). $P<0.05$, $n=3$. (c) Immunofluorescence analysis for CD15 on *Vastus Lateralis* muscles obtained from young and elderly donors. CD15 (green) signal detected outside the fibers, in an interstitial space position. Nuclei are counterstained with DAPI. Scale bars: 200 µm. In the right panel, the graph indicates the quantification of the number of $CD15^+$ cells per mm^2 of tissue. $P<0.05$, $n=3$. For a-c, two-tailed Student's t-test was used and results are displayed as mean ±SEM.

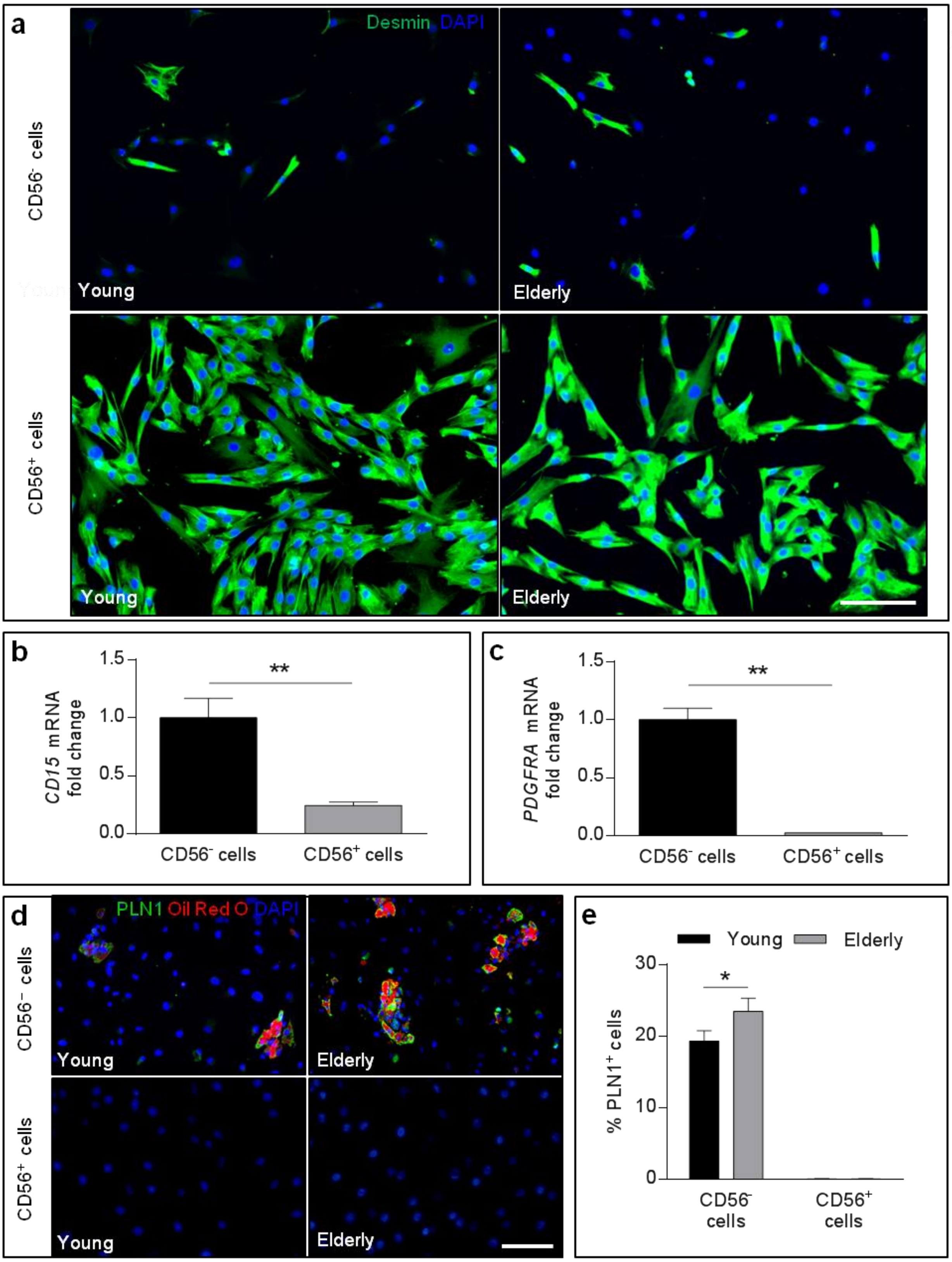

FIGURE S2 *(See legend on next page)*

(See figure on previous page)

FIGURE S2 Further characterization of human muscle-derived cells from young and elderly donors sorted for CD56. (a) Immunofluorescence analysis for desmin (green) in $CD56^{+}$/$CD56^{-}$ sorted cells from young and elderly donors. Nuclei are counterstained with DAPI. Scale bars: 200 µm. b, c) qRT-PCR analysis for *CD15* (b) and *PDGFRA* (c) in $CD56^{-}$ and $CD56^{+}$ cells. $^{**}P<0.01$, $n=3$ per group. (d) Immunofluorescence analysis of perilipin (PLN1, green) on $CD56^{-}$ and $CD56^{+}$ cells differentiated towards the adipogenic lineage for 14 days. Oil Red O staining can be observed in red channel. Nuclei are counterstained with DAPI. Scale bars: 200 µm. (e) Quantification of $PLN1^{+}$ cells from (d). $^{*}P<0.05$, $n=5$ per group. For b, c, e, two-tailed Student's t-test was used and results are displayed as mean± SEM.

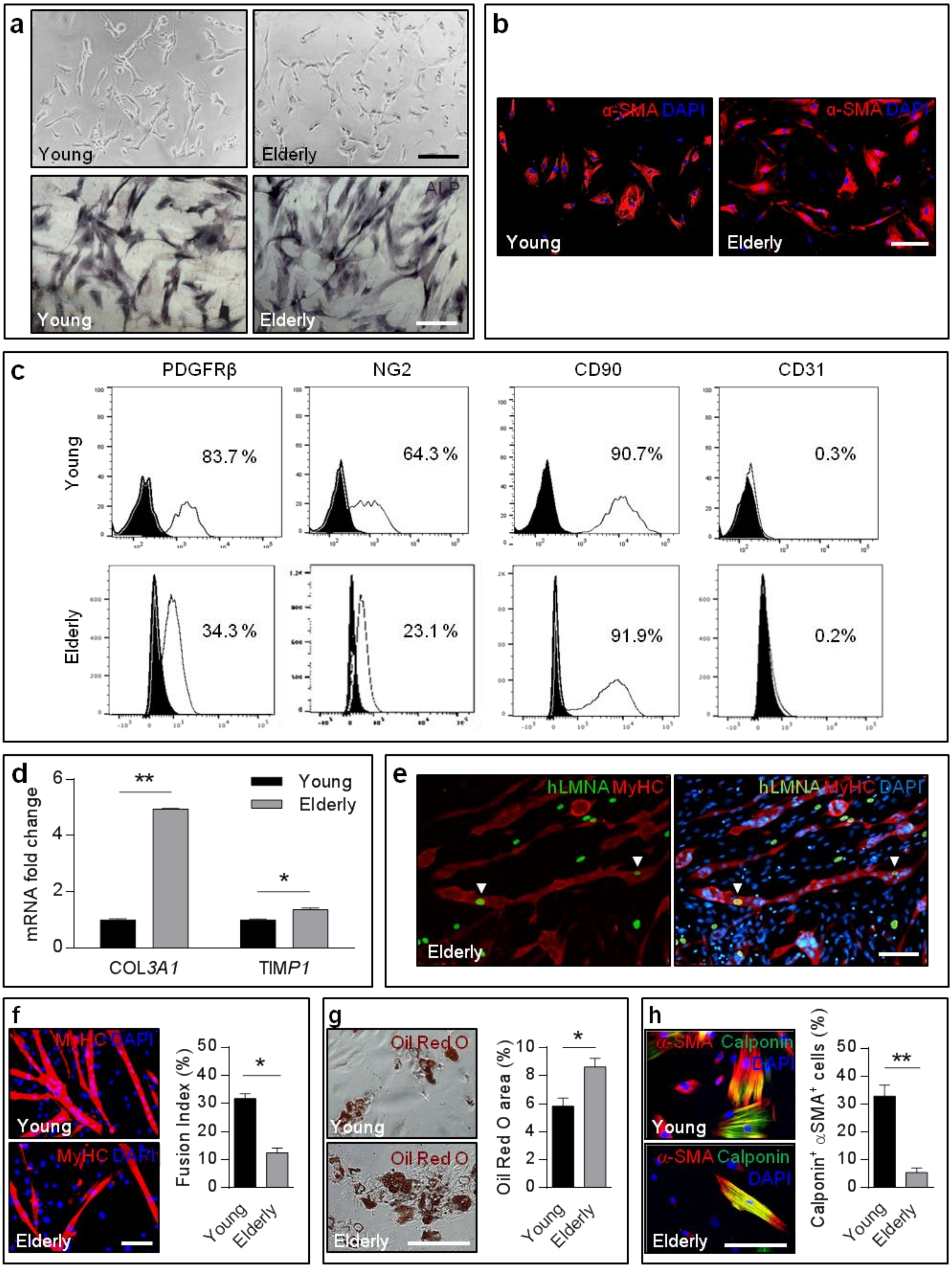

FIGURE S3 *(See legend on next page)*

(See figure on previous page)

FIGURE S3 Further characterization of young and elderly MABs. (a) Cell morphology (upper panels) and ALP enzymatic staining (lower panels) of sorted young and elderly MABs. Scale bars: 100 µm. (b) Immunofluorescence analyses for α-SMA (red) in young and elderly MABs. Nuclei are counterstained with DAPI (blue). Scale bars: 100 µm. (c) Flow cytometry analysis for PDGFRβ, NG2, CD90 and CD31 of young and elderly MABs. In the plot isotype controls are represented in black peaks. (d) qRT-PCR analysis for the fibrosis markers *COL3A1* and *TIMP1* in undifferentiated young and elderly MABs. *$P<0.05$, $n=3$ per group. (e) Immunofluorescence analysis for hLMNA (green) and MyHC (red) in elderly MABs in co-culture with mouse myoblast C2C12 cells differentiated to the myogenic lineage for 8-10 days. Nuclei are counterstained with DAPI. Scale bars: 200 µm. (f) Representative images of immunofluorescence analysis for the myogenic marker MyHC (red) on young and elderly MABs cultured for 10 days in myogenic medium. Nuclei are counterstained with DAPI (blue). Scale bars: 200 µm. The graph indicates the quantitative analysis of the number of nuclei inside myotubes expressing MyHC (fusion index). *$P<0.05$, $n=3$ per group. (g) Representative images of Oil Red O staining on young and elderly MABs cultured for 14 days in adipogenic medium. Scale bars: 200 µm. The graph indicates the quantification of Oil Red O area in percentage of coverage per field. *$P<0.05$, $n=3$ per group. (h) Representative images of immunofluorescence analysis for the smooth muscle markers calponin (green) and α-SMA (red) on young and elderly MABs cultured for 7 days in smooth muscle differentiation medium. Nuclei are counterstained with DAPI (blue). Scale bars: 200 µm. The graph indicates the quantitative analysis of the percentage of differentiated cells expressing both smooth muscle markers. **$P<0.01$, $n=3$ per group. For d, f-h, two-tailed Student's t-test was used and results are displayed as mean ±SEM.

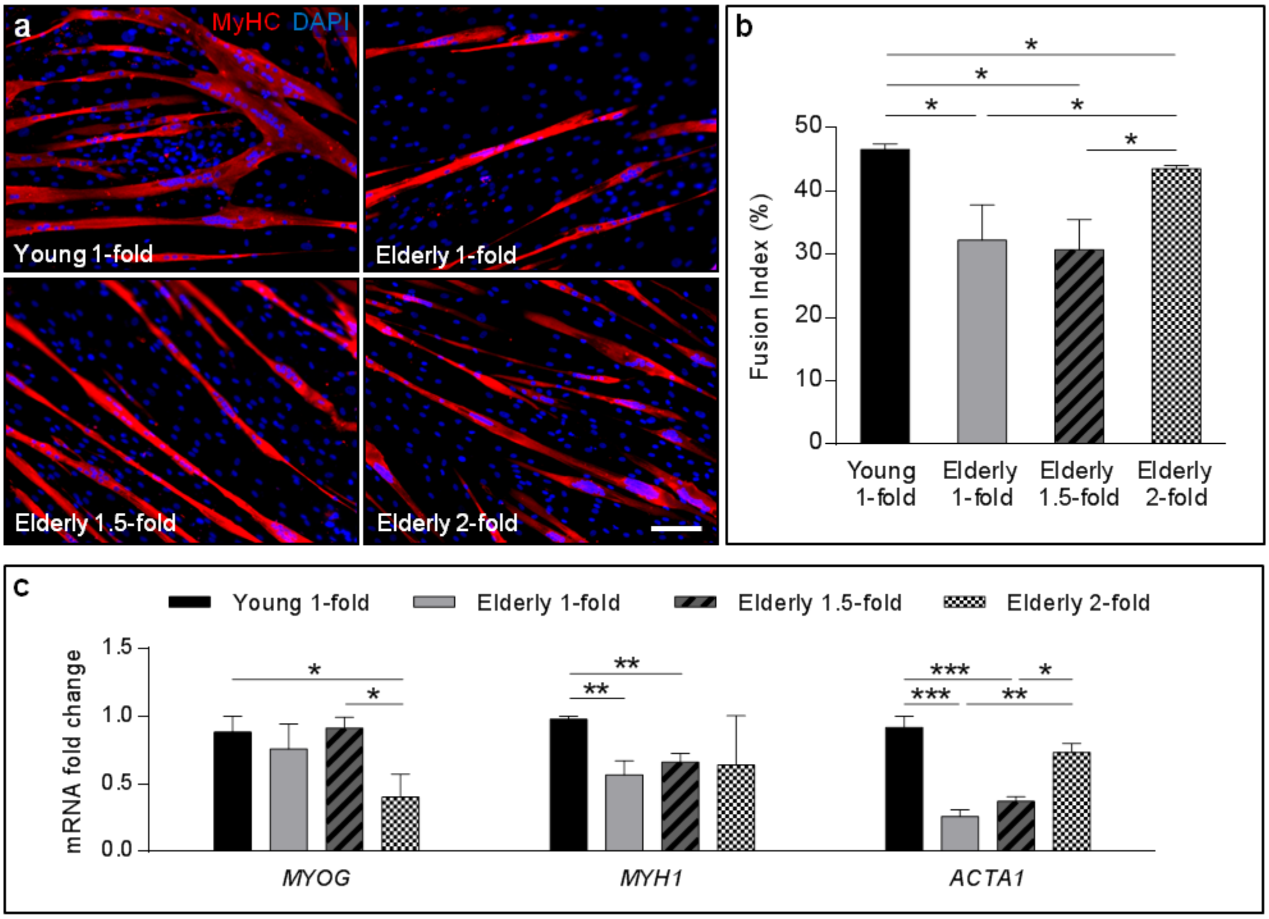

FIGURE S4 *In vitro* myogenic potential of elderly MABs seeded at different densities vs. young MABs. (a) Immunofluorescence analysis for MyHC (red) in young MABs and elderly MABs seeded at different densities after 10 days in myogenic medium. Nuclei are counterstained with DAPI (blue). Scale bars: 200 µm. (b) Quantification of the number of nuclei inside myotubes expressing MyHC (fusion index). *$P<0.05$, $n=3$ per group. (c) qRT-PCR analysis for the myogenic markers *MYOG*, *MYH1* and *ACTA1* in young MABs and elderly MABs seeded at different densities. **$P<0.01$, ***$P<0.001$, $n=3$ per group. For b, c, two-tailed Student's t-test was used and results are displayed as mean ±SEM.

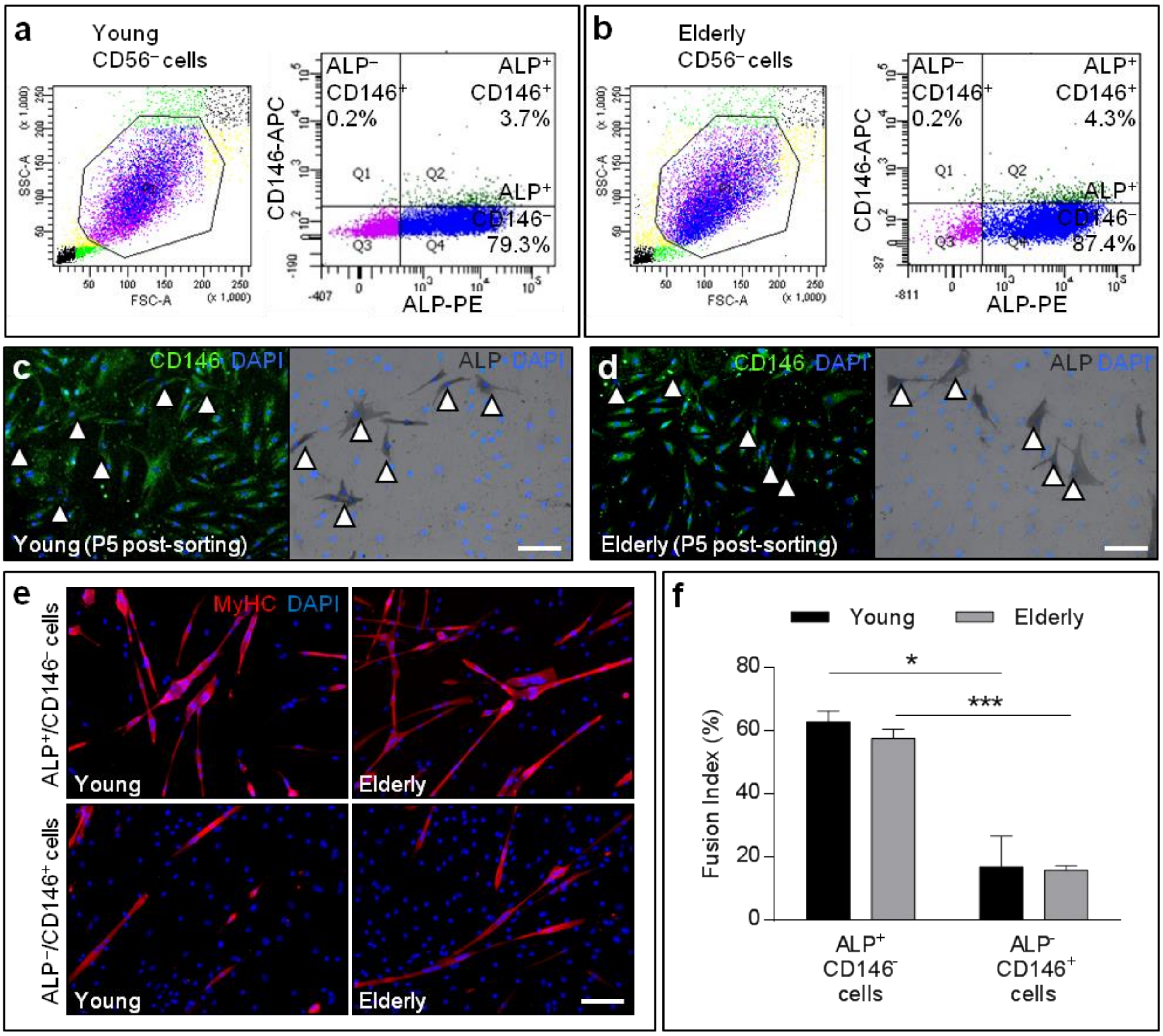

FIGURE S5 Fluorescence-activated cell sorting analysis and *in vitro* myogenic differentiation of CD56^{-}/ALP^{+}/CD146^{-} and CD56^{-}/ALP^{-}/CD146^{+} cells. (a, b) Examples of dot plot indicating the different subpopulations from young (a) and elderly (b) donors obtained by combining anti-ALP-PE and anti-CD146-FITC antibodies. (c, d) Immunofluorescence analysis for CD146 (green) and ALP immunochemistry staining (black) of sorted young (c) and elderly (d) CD56^{-}/ALP^{+}/CD146^{+} cellular fraction after 5 passages in culture. Arrowheads indicate double positive cells. (e) Immunofluorescence analysis for MyHC (red) of CD56^{-}/ALP^{+}/CD146^{-} and CD56^{-}/ALP^{-}/CD146^{+} cells after 10 days in myogenic medium. For c-e, nuclei are counterstained with DAPI; scale bars: 200 µm. (f) Quantification of the number of nuclei inside myotubes expressing MyHC in e. *P<0.05, ***P<0.001, n=3 per group. Two-tailed Student's t-test was used and results are displayed as mean± SEM.

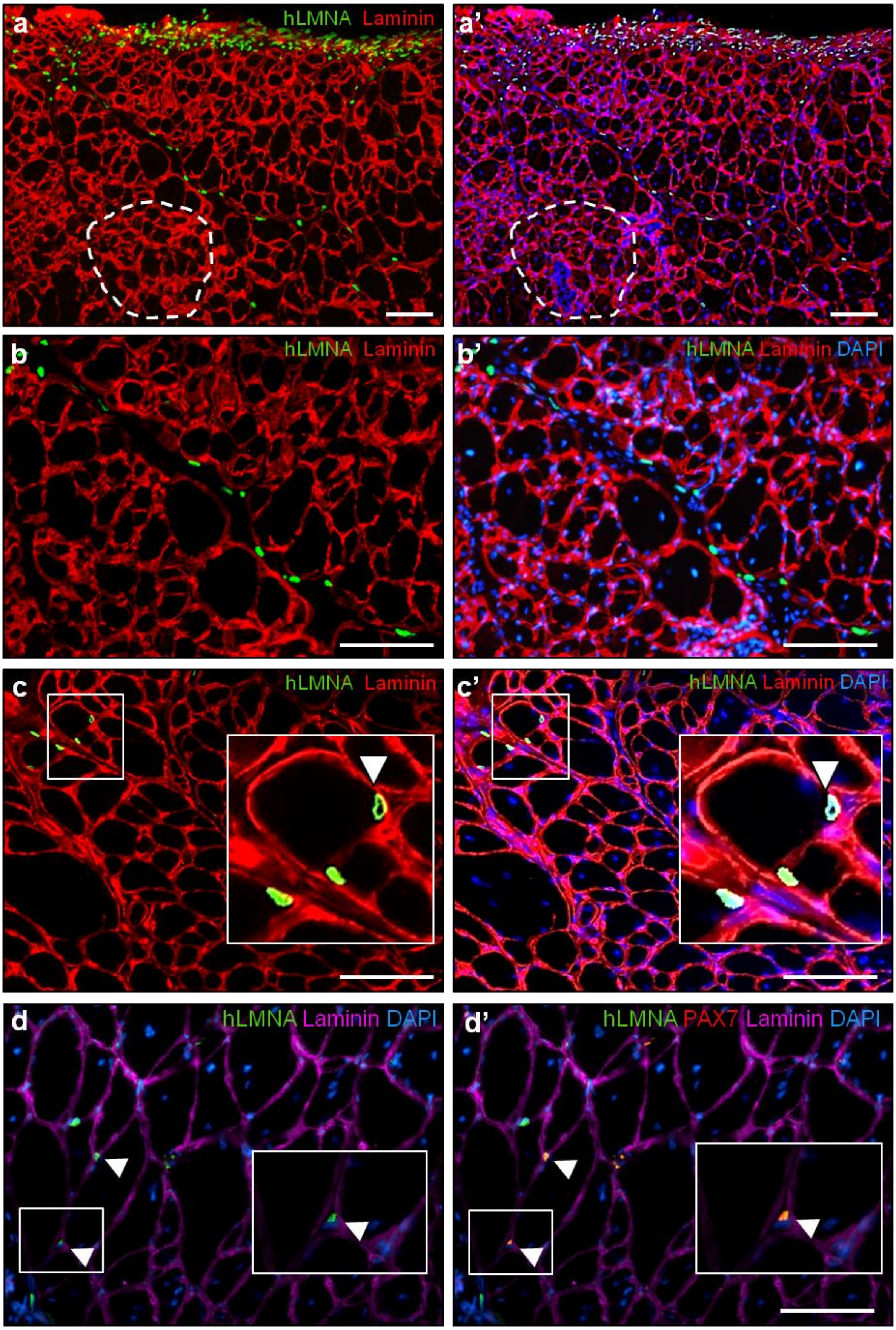

FIGURE S6: ***(****See legend on next page)*

(See figure on previous page)

FIGURE S6 *In vivo* engraftment and localization of MABs injected into TA muscles of *Sgcb-null Rag2-null γc-null mice.* (a-c') Immunofluorescence analysis for hLMNA (green) and laminin (red) in transplanted muscles with human MABs. MABs engrafted clearly in the muscles (a, a') and they were detected in the muscle interstitium (b, b'). Moreover, human nuclei were also localized in the satellite cell niche, between fibers and the basal lamina (c, c'). Enlarged magnification of area is shown in the insets. (d, d') Immunofluorescence analysis for hLMNA (green), laminin (purple) and PAX7 (red) in transplanted muscles with human MABs. For a-d and a'-d' nuclei are counterstained with DAPI (blue). Scale bars: 100 µm.

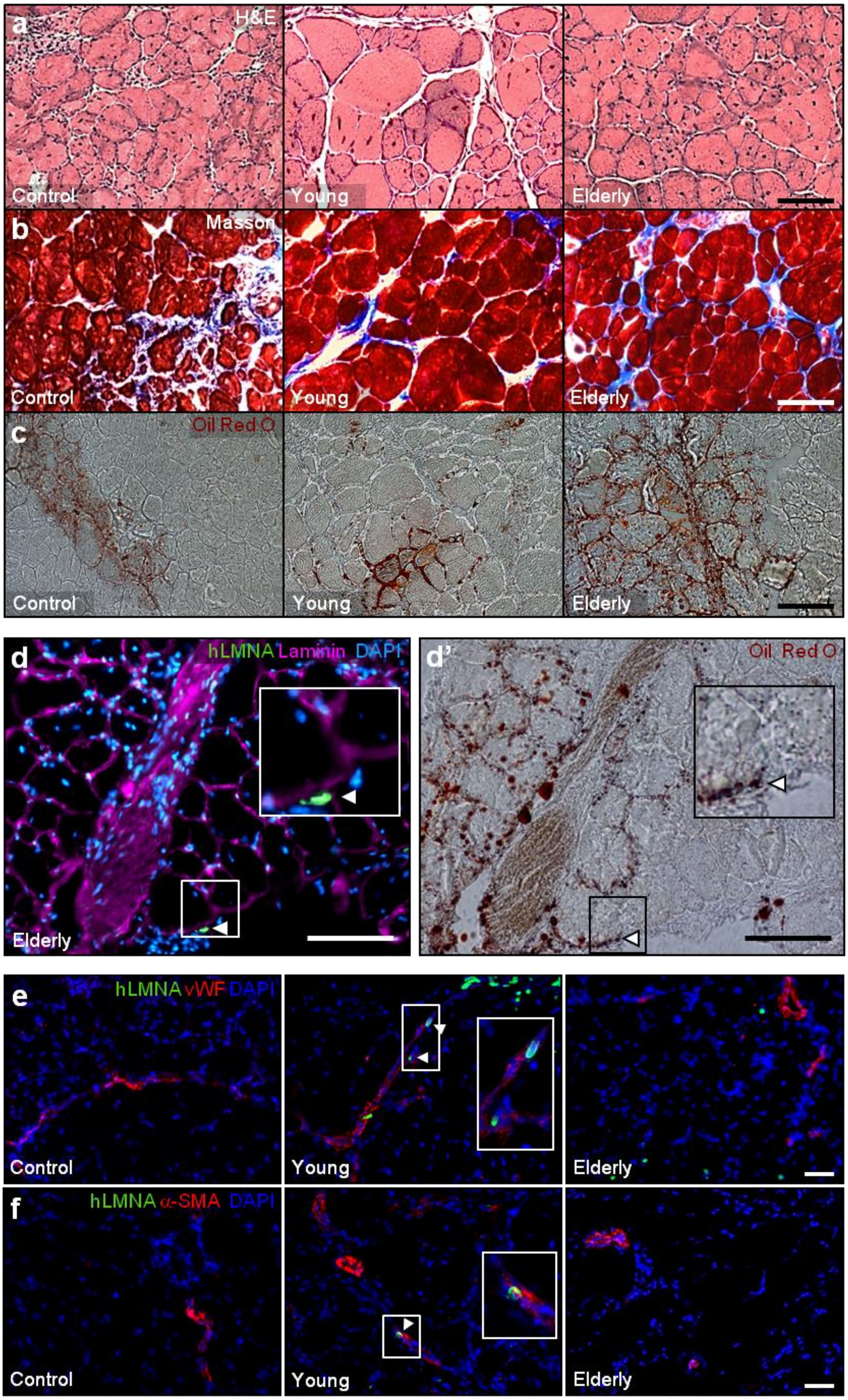

FIGURE S7 *(See legend on next page)*

(See figure on previous page)

FIGURE S7 Histological stainings for morphometric analyses of the TA muscles of *Sgcb-null Rag2-null yc-null* mice injected with young, aged MABs and MAB paracrine effects. (a) Representative images of Hematoxylin and Eosin staining of muscle sections injected with young and elderly MABs compared to uninjected muscles. (b) Representative images of Masson's trichrome staining of muscle sections injected with young and elderly MABs and uninjected muscles. (c) Oil red O staining on muscle sections injected with young and elderly MABs and uninjected muscles. (d, d') Immunofluorescence analysis for hLMNA (green), laminin (purple) and Oil Red O staining of the same section of muscle transplanted with elderly MABs. Arrowheads and enlarged areas show co-localization of a human nucleus with fat tissue. (e, f) Immunofluorescence analysis of hLMNA (green) in combination with the endothelial marker vWF (red, in e) or with the smooth muscle marker α-SMA (red, in f) in transplanted muscles with young and elderly MABs and uninjected muscles. Arrowheads and enlarged areas show integration of young MABs in the vessels. For d, e, f, nuclei are counterstained with DAPI (blue). For a-f, scale bars: 100 µm.
