# Supplementary material for: Aging affects the in vivo regenerative potential of human mesoangioblasts
Source: Aging Cell. 2018 Feb 4;17(2):e12714. doi: 10.1111/acel.12714 (PMC5847873; doi:10.1111/acel.12714)
Supplement: Supplementary file 2 [file ACEL-17-e12714-s002.docx]

## APPENDIX S1: Supporting information on the experimental procedures

## Participant enrolment

Human muscle samples were derived from *Vastus Lateralis* muscle biopsies using the Tiny Percutaneous Needle-Biopsy (TPNB) ([Pietrangelo *et al.* 2011](#_ENREF_29)). Young and elderly healthy untrained subjects underwent voluntary biopsies. All these subjects provided written informed consent before participating in the present study. This study was approved by the Ethics Committee for Biomedical Research, University of Chieti (PROT 1884 COET), and complied with the Declaration of Helsinki (as amended in 2000). The criteria for the selection were normal ECG, blood pressure, absence of metabolic, cardiovascular, chronic bone/joint and muscular diseases. Information about the age and the gender of subjects can be found in the table below (Table: S1)

| **Donor** | **Age** | **Gender** |
| --- | --- | --- |
| Young #1 | 20 years | Male |
| Young #2 | 37 years | Male |
| Young #3 | 22 years | Male |
| Young #4 | 35 years | Male |
| Young #5 | <35 years | Male |
| Elderly #1 | 48 years | Female |
| Elderly #2 | 72 years | Male |
| Elderly #3 | 82 years | Male |
| Elderly #4 | 66 years | Male |
| Elderly #5 | 64 years | Male |
| Elderly #6 | 80 years | Male |
| Elderly #7 | 81 years | Female |

Table S1: Information about the age and the gender of subjects can be found in the table below

supplementary data (Table S1).

## Fluorescence Activated Cell Sorting

For flow cytometry analyses and Fluorescence Activated Cell Sorter (FACS), cultured cells were detached by using 0.25% trypsin-EDTA, counted and resuspended in the appropriate volume of Flow Cytometry Staining Medium (0.1% BSA in PBS). Then, the primary antibody at the right dilution was added (see Table S2 below) and the cells were incubated for 30 minutes at 4ºC in the dark. After stopping the reaction with 1% BSA, cells were centrifuged, washed twice with PBS, filtered and resuspended in the appropriate volume of Staining Medium (≈250-300µl). For negative controls, cells were not stained with antibody or stained with an isotype control. FACS Canto machine (BD Biosciences) was used. Data were then charted by using FlowJo software (Tree Star, Ashland, OR, USA).

| **Antibody against** | **Company** | **Cat. Number** | **Dilution** |
| --- | --- | --- | --- |
| **CD56** | Biolegend | 304610 | 1:20 |
| **CD146** | Macs Milteny | 130-092-849 | 1:100 |
| **CD15** | BD Pharmigen | 555401 | 1:10 |
| **PDGFRα** | Abcam | ab119838 | 1:500 |
| **ALP** | R&D Systems | FAB1448P | 1:25 |
| **PDGFRβ** | Abcam | ab119861 | 1:500 |
| **NG2** | R&D | FAB2585P | 1:100 |
| **CD90** | Abcam | ab124527 | 1:200 |
| **CD31** | Ebioscience | 17-0319-41 | 1:20 |

Table S2: Primary antibodies used for FACS analyses.

## Immunofluorescence analyses

For *in vitro* analyses, cells were fixed with 4% paraformaldehyde for 15 minutes at room temperature and then, after 3 washes in PBS, permeabilized with 1% BSA in PBS + 0.2% Triton X-100 (Sigma) for 30-45 minutes at room temperature. Cells were then blocked for 30 minutes with 10% donkey serum at room temperature and after that incubated overnight at 4ºC with the primary antibody (for dilutions see Table S3 below). The following day, after 3 PBS washes, they were incubated for 1 hour at room temperature with the secondary antibody (Alexafluor®, Invitrogen) and washed again 3 times. DAPI 1:3000 was used, the cells were washed 3 times again and the immunofluorescence was mounted using FluorSave™ (Millipore).

For *in vivo* analyses, slides were fixed with 4% paraformaldehyde for 15 minutes or Ethanol-Acetone 1:1 for 4 minutes at room temperature and permeabilized with 1% BSA + 0.5% Triton X-100 (Sigma) for 30 minutes at room temperature. Cells were incubated sequentially 60 minutes with 10% donkey serum, 2h at room temperature or overnight at 4ºC with the primary antibody (depending on the antibody, Table S3) and 1 hour at room temperature with the secondary antibody. DAPI 1:3000 was used and slides were mounted using FluorSave™ (Millipore). During the procedure, the same washes as in the *in vitro* analyses were performed.

The cells/slides were examined by fluorescence microscopy using Nikon Eclipse Ti. Images were merged and edited using ImageJ software.

| **Antibody against** | **Company** | **Cat. Number** | **Dilution** |
| --- | --- | --- | --- |
| **CD15** | Genetex | GTX60470 | 1:300 |
| **Desmin** | Sigma | D8281 | 1:200 |
| **Ki67 (*in vitro*)** | BD Biosciences | 556003 | 1:300 |
| **MyHC** | Hybridoma Bank | - | 1:5 |
| **PLN1** | Sigma | P1873 | 1:200 |
| **α-SMA** | Sigma | C6198 | 1:200 |
| **hLMNA** | Novocastra | NCL-LAM-A/C | 1:100 |
| **CD146** | Abcam | ab24577 | 1:300 |
| **Calponin** | Abcam | ab46794 | 1:200 |
| **Laminin** | Sigma | L9393 | 1:300 |
| **PAX7** | Hybridoma Bank | - | 1:20 |
| **SGCB** | Novocastra | NCL-b-SARC | 1:75 |
| **Ki67 (*in vivo*)** | Thermo Scientific | RM-9106-S | 1:100 |
| **COL3** | Abcam | ab7778 | 1:400 |
| **vWF** | Abcam | ab6994 | 1:400 |

Table S3: Primary antibodies used for immunofluorescence analyses.

## Alkaline Phosphatase Enzymatic Staining

SIGMAFAST™ BCIP®/NBT (Sigma) for the detection of ALP activity was used following manufacturer’s instructions. Briefly, one tablet was dissolved in 10ml of water, and 1 ml was added in a 24-well containing CD56^-^ cells or MABs. The solution was kept for 2 hours at 37ºC. Fibroblasts were used as a negative control.

## qRT-PCR analyses

For quantitative Reverse Transcription Polymerase Chain Reaction (qRT-PCR) analyses, RNA was isolated through Purelink® RNA mini kit (Thermo Fisher Scientific), and gDNA traces were removed by Turbo™ DNase (Thermo Fisher Scientific). One µg RNA was reverse-transcribed using SuperScript™ III Reverse Transcriptase (Thermo Fisher Scientific). Then, quantitative Polymerase Chain Reaction was performed in 384-multiwell plates (10 μl final volume; thermal profile, 95 °C 15′′ −60 °C 45′′ (× 50)), using Platinum SYBR Green Mix (Thermo Fisher Scientific), 1 μl of 1:5 diluted cDNA and 250 nM primers (for primer sequences, see Table S4 below).

| **Gene** | **Forward** | **Reverse** |
| --- | --- | --- |
| ***ALP*** | ACTGGTACTCAGACAACGAGAT | ACGTCAATGTCCCTGATGTTATG |
| ***CD146*** | GGGTACCCCATTCCTCAA | CAGTCTGGGACGACTGAATG |
| ***CD15*** | GATCTGCGCGTGTTGGACTA | GAGGGCGACTCGAAGTTCAT |
| ***PDGFRA*** | TGGCAGTACCCCATGTCTGAA | CCAAGACCGTCACAAAAAGGC |
| ***PPARG*** | ACCAAAGTGCAATCAAAGTGGA | ATGAGGGAGTTGGAAGGCTCT |
| ***MYOD1*** | TGCTCCGACGGCATGATGGACTA | TTGTAGTAGGCGCCTTCGTAGCAGTT |
| ***CDKN2A*** | TCTTGCATCTCCACCAGCTG | CTCCAGGTTTCCCATTTAGC |
| ***IGFBP5*** | CGCGGGGTTTGCCTCAACGA | CTGCGGCAGGGGCCTTGTTC |
| ***CCNA*** | AGAGTGCACGCTGCTTGG | GTGCAACCCGTCTCGTCTTC |
| ***CCNE*** | CCTGTACTGAGCTGGGCAAA | TTTTGGCTGCAGAAGAGGGT |
| ***CDC6*** | GACACAAGCTACCATGGTTT | CAGGCTGGACGTTTCTAAGTT |
| ***MCM3*** | GAGCAAAGTTCGGGAGCTGA | TTGTTCAGAAGCCGGTTAGC |
| ***COL3A1*** | GGTGCTCGGGGTAATGAC | TCCAGGGAATCCGGCAGTT |
| ***TIMP1*** | CTTCTGCAATTCCGACCTCGT | CCCTAAGGCTTGGAACCCTTT |
| ***MYOG*** | GGGCCCCTGGAAGAAAAG | AGGAGGCGCTGTGGGAGTT |
| ***MYH1*** | CTCTTCCCGCTTTGGTAAGTT | CAGGAGCATTTCGATTAGATCCG |
| ***ACTA1*** | GCTCTTCCAGCCTTCCTTTAT | CATACAGGTCCTTCCTGATGTC |
| ***GAPDH*** | TCAAGAAGGTGGTGAAGCAGG | ACCAGGAAATGAGCTTGACAAA |

Table S4: Primers used for qRT-PCR analyses.

##

## In vitro skeletal muscle differentiation

Cells from different donors were differentiated to skeletal muscle using differentiation medium consisting of DMEM-high glucose (Life Technologies) supplemented with 2% horse serum (Life Technologies), 1% Sodium Pyruvate (Life technologies), 1% Glutamine (Sigma) and 1% P/S (Life technologies). The cells were plated in 12-well plates at a density of 2x10^4^ cells/cm^2^ and induction was started when confluence of 85% was reached. Medium was changed every 2-3 days for 8-10 days.

## Culture of C2C12 cells

Mouse myoblast cell line C2C12 was cultured with DMEM 4.5 g/L glucose supplemented with 10% FBS (Sigma), 1% Glutamine (Sigma), 1% Sodium Pyruvate (Life Technologies) and 1% Penicillin/Streptomycin (Life Technologies). For co-culture experiments, C2C12 cells and MABs were plated at 1:3 ratio in 12-well plates at a density of 2x10^4^ cells/cm^2^ and induced using the described skeletal muscle differentiation medium. Medium was changed every 2-3 days for 8 days.

## In vitro adipogenic differentiation

Cells from different donors underwent adipogenic differentiation using StemPro® Adipogenesis Differentiation Kit (Gibco). Briefly, the cells were plated in 12-multiwell plates at a density of 2x10^4^ cells/cm^2^ and induction was started when the right confluence was reached. Medium was changed every 2-3 days for 14 days.

## Oil Red O Staining

To stain lipids, 4% paraformaldehyde fixed cells or tissues were rinsed twice with PBS and then incubated with Oil red O Solution for 15-30 minutes at room temperature. After that, the Oil Red O Solution was removed and cells were washed three times with water. Images were acquired with the microscopy Nikon Eclipse Ti and edited using the ImageJ software. Fields reported in the figures are representative of all examined fields.

## In vitro smooth muscle differentiation

MABs from different donors were differentiated to smooth muscle using differentiation media consisting of High Glucose DMEM (Life Technologies) supplemented with 2% Horse Serum (HS, Life Technologies), 1% Sodium Pyruvate (Life technologies), 1% Glutamine (Sigma), 1% Pen-Strep (Life technologies) and 50ng/ml transforming growth factor β1 (TGF-β1, Peprotech). Cells were plated in 12-well plates at 1x10^4^ cells/cm^2^ and differentiation was started when they reach 70% confluence. Medium was refreshed every 2-3 days for 8 days.

## Crystal Violet Staining

0.05% (w/v) crystal violet staining solution was prepared by dissolving 40 mg of crystal violet powder (Sigma) in 80 mL of Milli-Q water (Millipore). Petri dishes with MAB colonies at day 14 after seeding were fixed using 4% paraformaldehyde for 10-15 minutes at room temperature. After washing with PBS, 0.05% crystal violet staining solution was added and incubated for 20 minutes at room temperature. Subsequently petri dishes were washed twice with Milli-Q water. The images were acquired using Nikon Eclipse Ti Microscope and NIS-Elements AR 4.11 Software.

## Hematoxylin and Eosin Staining

Paraffin-embedded sections were deparaffinized and rehydrated. Cryosections were thawed and fixed with 4% paraformaldehyde for 10-15 minutes. Then, muscle sections were stained in Harris hematoxylin for 4 minutes and washed afterwards in running tap water for 2 minutes. After that, the sections were subsequently soaked for 1 minute each in acid alcohol, running tap water, bluing reagent, running tap water, eosin, 95% ethanol, 100% ethanol and HistoClear. The slides were mounted and left on a slide heater overnight. The staining was then observed and images acquired using Nikon Eclipse Ti Microscope and NIS-Elements AR 4.11 Software.

## Masson’s Trichrome Staining

To stain fibrotic tissue Masson's trichrome analysis was used. Prior starting, paraffin-embedded sections were deparaffinized to deionized water and preheated in Bouin’s Solution. Cryosections were thawed and fixed with 4% paraformaldehyde for 10-15 minutes. Then, muscle sections were stained in Working Weigert's Iron Hematoxylin Solution for 5 minutes, washed in running tap water for 5 minutes and stained in Biebrich Scarlet-Acid Fucshin for 5 minutes. After that, slides were rinsed in de-ionized water, placed in Working Phosphotungstic\Phosphomolybdic acid solution for 5 minutes, stained in Aniline Blue solution for 5 minutes and then in Acetic acid 1% for 2 minutes. At the end, slides were mounted and left in a slide heater overnight. Images were acquired with the microscopy Nikon Eclipse Ti and edited using the ImageJ software. Fields reported in the figures are representative of all examined fields.

## Statistical analyses

Data from the different experiments were analyzed using the program GraphPad Prism. Two-tailed Student’s test or one-way ANOVA were used to compare interrelated samples. Confidence intervals were fixed at 95% (p<0.05), 99% (p<0.01) and 99.9% (p<0.001). GraphPad Prism was also used to graph the results as the average ± standard error of the mean (SEM) (see figure legends for specific information regarding the number of independent experiments or biological replicates).
